# Global positioning system-based food environment exposures, diet-related, and cardiometabolic health outcomes: a systematic review and research agenda

**DOI:** 10.1186/s12942-024-00362-x

**Published:** 2024-02-06

**Authors:** Noreen Z. Siddiqui, Lai Wei, Joreintje D. Mackenbach, Maria G. M. Pinho, Marco Helbich, Linda J. Schoonmade, Joline W. J. Beulens

**Affiliations:** 1 Epidemiology and Data Science, Amsterdam UMC, Vrije Universiteit Amsterdam, De Boelelaan 1089a, 1081 HV Amsterdam, The Netherlands; 2 Amsterdam Public Health, Health Behaviors and Chronic Diseases, Amsterdam, The Netherlands; 3 Department of Human Geography and Spatial Planning, Utrecht University, Utrecht, The Netherlands; 4 Upstream Team, Amsterdam, the Netherlands; 5 Copernicus Institute of Sustainable Development, Utrecht University, Utrecht, The Netherlands; 6 Medical Library, Vrije Universiteit Amsterdam, Amsterdam, The Netherlands; 7 Julius Center for Health Sciences and Primary Care, University Medical Centre Utrecht, Utrecht, The Netherlands

**Keywords:** Global Positioning Systems, Food environment, Cardiometabolic health

## Abstract

**Background:**

Geographic access to food may affect dietary choices and health outcomes, but the strength and direction of associations may depend on the operationalization of exposure measures. We aimed to systematically review the literature on up-to-date evidence on the association between food environment exposures based on Global Positioning System (GPS) and diet-related and cardiometabolic health outcomes.

**Methods:**

The databases PubMed, Embase.com, APA PsycInfo (via Ebsco), Cinahl (via Ebsco), the Web of Science Core Collection, Scopus, and the International Bibliography of the Social Sciences (via ProQuest) were searched from inception to October 31, 2022. We included studies that measured the activity space through GPS tracking data to identify exposure to food outlets and assessed associations with either diet-related or cardiometabolic health outcomes. Quality assessment was evaluated using the criteria from a modified version of the Newcastle–Ottawa Scale (NOS) for cross-sectional studies. We additionally used four items from a quality assessment tool to specifically assess the quality of GPS measurements.

**Results:**

Of 2949 studies retrieved, 14 studies fulfilled our inclusion criteria. They were heterogeneous and represent inconsistent evidence. Yet, three studies found associations between food outlets and food purchases, for example, more exposure to junk food outlets was associated with higher odds of junk food purchases. Two studies found associations between greater exposure to fast food outlets and higher fast food consumption and out of three studies that investigated food environment in relation to metabolic outcomes, two studies found that higher exposure to an unhealthy food environment was associated with higher odds of being overweight.

**Conclusions:**

The current and limited evidence base does not provide strong evidence for consistent associations of GPS-based exposures of the food environment with diet-related and cardiometabolic health outcomes.

**Supplementary Information:**

The online version contains supplementary material available at 10.1186/s12942-024-00362-x.

## Background

Globally, cardiovascular diseases and type 2 diabetes are leading causes of morbidity and mortality and their prevalence is expected to increase [1, 2]. Lifestyle factors such as unhealthy dietary patterns and physical inactivity are major risk factors [3, 4]. The environment that individuals are exposed to in daily life likely contributes to these diseases [5–7]. One central aspect of daily living environments that has changed drastically over the past decades is the food environment [8]. As such, an unhealthy food environment is hypothesized to be an important upstream risk factor of cardiometabolic diseases [9]. Indeed, the number and type of food retailers that individuals are exposed to is likely to influence their food choice behaviors and dietary intake [9]. Despite this, studies so far have observed inconsistent associations between exposure to the food environment and dietary behaviors and diet-related risk factors [10–12].

There are different approaches to measure people’s exposure to food environment, including proximity to a nearest shop or restaurant or the density of food retailers present [13, 14]. The uncertainty in the exposure assessment may be one explanation for the inconsistent evidence [10–12]. Most previous studies have focused on the residential food environment, ignoring the fact that individuals spend a considerable proportion of their time outside the residence in settings such as the workplace or sports club [10, 15–17]. Previous studies indeed showed that, for example, the work environment is an important contributor to exposure to fast food outlets which may deviate from the outlets where people reside [18–20]. Therefore, only measuring exposures at residential locations may yield inaccurate estimates of exposure to the food environment. The multiple places at which individuals interact with or are exposed to the food environment can be represented through their ‘activity space’ (i.e., subsuming people’s daily travel patterns and the locations visited [21, 22]). While surveys and travel diaries are prone to inaccuracies due to possible recall biases, the use of Global Positioning System(s) (GPS) to define exposure to the food environment is the most accurate method to capture an individual’s day-to-day activity space objectively [22–31]. GPS-based food environment studies capture the out-of-home locations and routes individuals visit, thus more precisely capturing the totality and the duration of exposure to the food environment.

A systematic review dating from 2016 identified studies that investigated exposure to the food environment by using GPS tracking in relation to diet or other health outcomes [32]. However, this systematic review included only six articles, of which only four investigated associations with diet or health outcomes [32], and new studies have been published since then. Therefore, we aimed to systematically review the literature on up-to-date evidence on the association between food environment exposures based on GPS and diet-related and cardiometabolic health outcomes.

## Methods

### Review design

This systematic review was reported in accordance with the Preferred Reporting Items for Systematic Reviews and Meta-Analysis (PRISMA) statement [33]. The protocol for this systematic review was registered before the literature search in the PROSPERO database (registration number: CRD42022343431).

### Data sources and search strategy

In collaboration with a medical information specialist (LS), a comprehensive systematic search was performed in the bibliographic databases PubMed, Embase.com, APA PsycInfo (via Ebsco), Cinahl (via Ebsco), the Web of Science Core Collection, Scopus, and the International Bibliography of the Social Sciences (via ProQuest) from inception to October 31, 2022. While Global Positioning System (GPS) is a subset of the Global Navigation Satellite System (GNSS), encompassing all satellite navigation systems worldwide, we will use the term ‘GPS’ in the search strategy and throughout this article, since this term is most frequently used. Search terms included controlled terms as well as free text terms. Synonyms for ‘food environment’ (e.g., ‘community food environment’) were combined with terms for ‘GPS-exposure’ (e.g., ‘Global Positioning System’) and ‘diet-related outcomes’ (e.g., ‘diet quality’, ‘metabolic syndrome’). We additionally performed a search in Google Scholar to check for additional references. The full search strategies for each database are reported in the Additional file 1: Tables S1a–g. The search was performed without date or language restrictions. Duplicate articles were excluded using Endnote X20.0.1 (Clarivate™), following the Bramer-method [34].

### Inclusion and exclusion criteria

Studies were included if they met the following criteria: (1) included humans (all ages) in their study population; (2) used GPS-enabled devices (e.g., smartphones, GPS trackers) to identify the individuals’ exposure to food outlets; and (3) assessed diet-related (e.g., diet quality, food purchases or intake) or cardiometabolic health outcomes (e.g., hypertension, Body Mass Index (BMI)). We excluded studies for the following reasons: (1) did not measure exposures based on activity spaces, or defined activity spaces through non-GPS data (e.g., survey data); (2) did not report original scientific research (e.g., letters, conference abstracts, interviews, editorials, dissertations). Studies were not excluded if written in other languages than English.

### Study selection and data extraction

Four of the authors (NZS, LW, MH, JDM) performed a pilot test and screened the first hundred search results based on the title and abstract independently. No adaptation of the search string was needed after this pilot screening. Subsequently, two reviewers (NZS and LW) independently screened all potentially relevant titles and abstracts according to the eligibility criteria using Rayyan, a web-application designed to facilitate the initial title and abstracts’ screening in a systematic review [35]. Disagreements on in- or excluded articles were discussed and resolved among four of the authors (NZS, LW, MH, JDM). Full-text screening was performed independently by two authors (NZS and LW) to check eligibility of the included studies from the previous round. Again, disagreements were discussed and resolved among four of the authors (NZS, LW, MH, JDM).

Data from each included study was then extracted by NZS and LW and checked by JDM and MH for the following information: (1) reference; (2) country/location where the study was conducted; (3) percentage of female participants; (4) participant age range; (5) recruitment of study population; (6) sample size; (7) study design; (8) methods of GPS data collection (e.g., GPS trackers, mobile devices); 9) whether the studies reported loss of signal from GPS devices; (10) units of food environment exposure assessment based on GPS data (e.g., GPS point buffers); (11) tracking duration and GPS sampling frequency; (12) whether temporal aspects were taken into account (e.g., opening hours of food retailers); 13) and type and distribution of outcome measures (continuous/dichotomous, type of diet and/or cardiometabolic health outcome) (Additional file 1: Table S2).

### Quality assessment

Two authors (NZS and LW) independently evaluated the methodological quality of the included full text papers using the criteria from the Newcastle–Ottawa Scale (NOS) for cross-sectional studies, since all included papers had a cross-sectional study design [36]. The NOS estimates the risk of bias based on seven items which is divided into three categories: selection, comparability, and outcome [36]. Selection included the following items: representativeness of the samples, sample size calculation, and non-respondents. Comparability included the following items: ascertainment of the exposure (risk factor) and adjustment for confounders. Outcome included the following items: assessment of the outcome and whether the statistical test that was used was clearly described. The total points for each category were the following: four for selection, three for outcomes, and two for comparability (Additional file 1: Table S3). To date, there is no scoring system developed for the adjusted quality assessment tool of NOS to interpret the results. Therefore, we defined low quality studies as those who received less than 50% of all possible points to at least identify those studies with a high risk of bias.

In addition, we used four items from the quality assessment tool previously used by Cetateanu et al. [32], because these items specifically assess the quality of GPS measurements of the food environment which is not captured by the NOS. These were: (1) recording period, with scores ranging from 0 to 2 where zero was given for a recording period of ≤ 2 days, one for 3–4 days, and two for > 4 days); (2) assessment of variety of food outlet types, with scores ranging from 1 to 3, where one was given for one food outlet type, two for 2–4 food outlet types, and three for ≥ 5 food outlet types; (3) reporting of positional accuracy of the device used and whether GPS data quality was discussed. Scores from the latter items ranged from 0 to 1, where zero was given when positional accuracy was not reported and one when it was reported and scores ranging from 0 to 1, where zero was given when data quality was not discussed and one when data quality was discussed (Additional file 1: Table S3). The total score for these four items ranged from three to six. Since this tool has not been validated yet and only four items were used, we decided to present the scores in an Additional file 1: Table S3 and did not take the rating of these four items into account for our final rating. The quality assessment was performed individually by two authors (NZS and LW) and differences in judgement were discussed and resolved with JDM and MH.

### Data synthesis

Due to the heterogeneity of the included studies (e.g., small number of studies included, different population characteristics, differences in exposure, and outcome assessment), we were not able to pool effect sizes by means of meta-analyses or use any other synthesis method advised by Cochrane [37]. We, therefore, performed a narrative synthesis.

## Results

### Search outcome

**Fig. 1:**
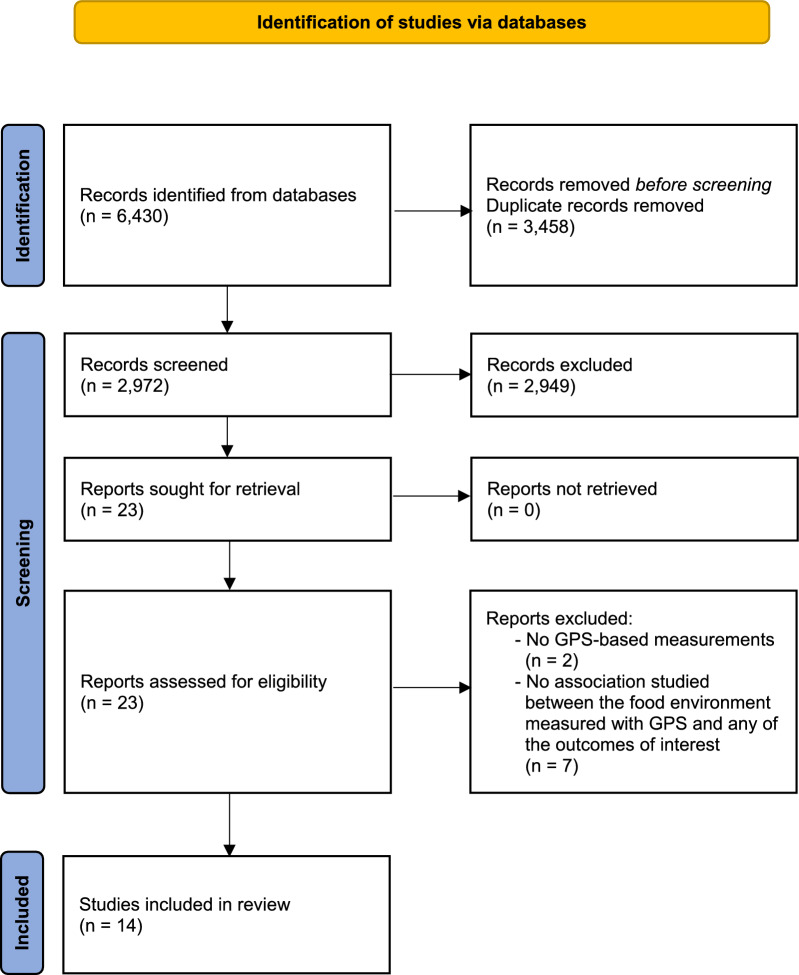
Flowchart of the search and selection procedure of the studies

Our search strategy identified 2972 articles from the databases after removing duplicates. Of these, 2949 articles were excluded because these were ineligible for full-text screening. We included 23 articles for full-text screening of which we excluded nine. Two studies used other measures than GPS to measure the food environment (e.g., questionnaires) and seven studies did not report on associations between the GPS-based food environment or the outcome of interest. Finally, we included 14 articles in the systematic review (Fig. 1).

We applied alphabetical references to cite the studies that have been included, a comprehensive list of these references can be found in Additional file 1.

### Quality assessment

Three studies scored less than 50% and were therefore defined as low quality studies^b,e,f^ (Table 1**)**. These studies mainly lacked information regarding the representativeness of the samples, sample size calculation, non-respondents, or performed low on the statistical testing. When additionally assessing GPS-based criteria (Additional file 1: Table S3), only one study reported the positional accuracy of the reported device^d^ and seven studies reported insufficient GPS-related information^a,c,f−i,k^.

**Table 1:** Quality assessment according to the New Ottowa Scale (NOS)

First author (year)	Selection				Comparability	Outcome		Total
	Representativeness of the samples	Sample size calculation	Non-respondents	Ascertainment of the exposure (risk factor)	Adjustment for confounders	Assessment of the outcome	Statistical test	
Widener (2018)^a^	0	1	0	1	1	1	1	5
Wray (2021)^b^	0	0	0	1	1	1	1	4
Sadler (2016)^c^	0	1	0	1	2	1	1	6
Shearer (2015)^d^	0	1	0	1	2	1	0	5
Zenk^e^	1	0	0	1	1	1	0	4
Ellistion (2020)^f^	1	1	0	1	0	1	0	4
Gustafson (2013)^g^	1	0	0	1	1	1	1	5
Ghosh Roy (2019)^h^	0	1	0	1	1	1	1	5
Seto (2016)^i^	1	1	0	1	2	1	1	7
Liu (2020)^j^	0	0	1	1	1	1	1	5
Christian (2012)^k^	1	0	1	1	1	1	1	6
Burgoine (2015)^l^	0	0	0	1	1	2	1	5
Tamura (2018)^m^	0	1	1	1	1	2	1	7
Wang (2018)^n^	1	0	0	1	1	1	1	5

### Study characteristics

The included articles were from the United States of America (USA) (n = 7)^e,g,h,k−n^, Canada (n = 5)^a−d,j^, Australia (n = 1)^f^, and China (n = 1)^i^ (Table 2**)**. All included studies had a cross-sectional study design. Food environment in these studies were assessed via GPS devices (n = 9) or via smartphone applications featuring GPS (n = 5). Loss of GPS signal (due to e.g., skyscraper buildings, wooded areas), was reported by five papers^(d,e,l,m,n)^ of which one study described this in the methods^(m)^, two studies accounted for this^(d,e)^, and the remaining papers mentioned this as a limitation in their study^(l,n)^. Tracking duration differed between the included studies, with most studies allowing a tracking period of 3 days (n = 2), six days (n = 6), or 2 weeks (n = 2).

**Table 2:** Study characteristics of the included studies

First author (year)	Country	Study population	% female and age range	Sample size	Methods of exposure data collection^(a)^	Food environment assessment based on GPS data^(b)^	Tracking duration and frequency of GPS sampling^(c)^	Temporal aspects^(d)^	Type and distribution of outcome^(e)^
Widener 2018^a^	Canada	(Young) Adults	66.5 16–30 years	496	Smartphone: CFSMobile app	Food environment data source: 2016 DMTI business directory; Food environment assessed: the number of grocery stores, convenience stores, fruit and vegetable markets, limited-service restaurants and ‘all food retailers’; GPS-based food exposure measures: regular activity space locations (defined as the top 1% and 10% of the time-weighted kernel density estimate surface)	7 days Frequency not reported	Not reported	Webform (Counts food purchasing)
Wray (2021)^b^	Canada	High school students	63.6 13–18 years	154	Smartphone: SmartAPPetite	Food environment data source: lists of food store places from the government; Food environment assessed: standardized count of logged GPS points within the buffer of retail food outlets's ads (quick service, restaurants, grocery, and variety); GPS-based food exposure measures: Euclidean buffers around outlets (150 m), billboards (150 m), bus shelters (75 m), and street posters (75 m)	12 weeks Every 120 s or when a user enters the geofenced area surrounding a retail food outlet	Not reported	Food purchases, categorical (quick service purchases, restaurants purchases, grocery purchases, variety purchases, all types of purchases)
Sadler (2016)^c^	Canada	Students	58.7 9–13 years	654	GPS device	Food environment data source: lists of food store places from public health inspectors; Food environment assessed: the number of minutes during which a child was exposed (i.e., within 50 m) to fast food, variety stores, pizza places or ice cream shops (ranges from 0 s to 350 min); GPS-based food exposure measures: 50 m buffers on food outlets	2 weeks Every second	Not reported	Junk food purchases outcome (binary), indicating whether junk food was purchased or not on the trip. Junk food was considered unhealthy food items purchased from fast food or variety stores, pizza places and ice cream shops
Shearer (2015)^d^	Canada	Students	Not reported 12–16 years	380	GPS device (20 channel EM-408 SiRFstar III chipset GSP receiver)	Food environment data source: lists of food store places from a commercial company (DMTI); Food environment assessed: The number of fast food (major fast food chains with a focus on take-away meals), restaurants (all other types), grocery and convenience stores, and average distances to every accessible and actually visited food locations within GPS route buffers from a participant's home and school origin; GPS-based food exposure measures: 50 m GPS route buffer	7 days Every second	Not reported	diet quality index ranging from 0 to 100 with higher scores reflecting better diet quality
Zenk (2011)^e^	USA	All ages	75.0 < 45, 45–64, > 64 years	120	GPS device: Foretrex 201	Food environment data source: lists of food store places from the government; Food environment assessed: fast food outlet density and the number of chain full-service grocery stores or supercenters; GPS-based food exposure measures: one standard deviation ellipse on GPS points and 0.5-mile daily path area on GPS points	7 days 30 s intervals	Not reported	Set of Food frequency questionnaire items reporting on saturated fat intake, fruit and vegetable intake, and whole grain intake (validation of FFQ not reported)
Elliston (2020)^f^	Australia	Adults	71.0 18 + years	72	Smartphone	Food environment data source: lists of food store places from the government; Food environment assessed: The number of food outlets; GPS-based food exposure measures: 50 m GPS point buffer	2 weeks Once the user report food intake	Not reported	Eating/Non-eating and food intake (collected by food reports and EMA measures: prompts, 4–5 times a day during a period of 2 weeks
Gustafson (2013)^g^	USA	Adults	58.0 18 + years	121	GPS device (Qstarz BT-Q1000XT Travel Recorder)	Food environment data source: lists of food store places from a commercial company (InfoUSA); Food environment assessed: the retail food environment index as a ratio of healthy (Supermarkets/grocery stores, farmers’ markets, and produce stands) relative to unhealthy (supercenters, convenience stores, fast-food restaurants, and gas stations with convenience stores, or less healthy venues) food venues; GPS-based food exposure measures: 0.5 mile GPS route buffer	3 days (2 weekdays and 1 weekend day) Frequency not reported	Not reported	Based on the NEMS-S protocol, availability, price, and quality of food were collected for 15 food categories (fruit, vegetables, milk, cheese, meat, baked goods, chips, beverages, canned items, cereal, desserts, prepared food items, snack foods, frozen meals, and beans) and 55 unique food items were assessed
Ghosh Roy (2019)^h^	USA	African American Women	100 25–65 years	79	GPS device (Qstarz BT-Q1000XT GPS, Qstarz)	Food environment data source: lists of food store places from a commercial company (Dun & Bradstreet); Food environment assessed: The number of fast food restaurants and convenience stores; GPS-based food exposure measures: 400 m GPS route buffer	7 days Every minute	Not reported	Snack food item and sweetened beverage intake dichotomized into non or one (0) or more than one (1)
Seto (2016)^i^	China	Students	66.7 18–31 years	12	Smartphone: CalFit Chi and Dong	Food environment data source: lists of food store places from Google Maps; Food environment assessed: average number of bakeries, bar, cafe, convenience store, food, grocery or supermarket, liquor store, meal delivery, meal takeaway, and restaurant; GPS-based food exposure measures: 250 m circular buffers on activity locations	6 days Every 10 s	Not reported	Portion size was recorded with video. Later, two trained dietitians familiar with local diets review the contents of the videos, and code the portion sizes and food groups associated with each food consumed. Subjects’ diet recordings were coded by both dietitians to assess inter-rater reliability
Liu (2020)^j^	Canada	(Young) Adults	65.0 16–30 years	591	Smartphone: Itinerium	Food environment data source: lists of food store places from opensource platform (OpenStreetMap); Food environment assessed: the number of fast food outlets, and the proportion of the sum of the number of fast food outlets, supermarkets, green groceries, and convenience stores within each activity space; GPS-based food exposure measures: 500 m, 1000 m, and 1500 m circular buffers on activity locations	7 days Frequency not reported	Time-weighted exposure was considered by calculating the proportion of the time a participant spent at each activity location in the total time spent in all activity space	Self-reported fast-food intake
Christian (2012)^k^	USA	Adults	56.4 18–65 years	121	GPS device (Qstarz BT-Q1000XT Travel Recorder)	Food environment data source: lists of food store places from the government; Food environment assessed: the retail food environment index calculated as the sum of fast food restaurants plus convenience stores, divided by the sum of supermarkets plus fruit/vegetable markets, the proportion and density of limited-service outlets and supermarket; GPS-based food exposure measures: 0.5 mile GPS route buffer	3 days Every 3 s	Not reported	Continues dietary intake of added sugar, red meat, fried potatoes, fruits and vegetables, whole grains. Weight status categorized into overweight and obese
Burgoine (2015)^l^	USA	Children	48.9 5–11 years	94	GPS device (Qstarz BT-Q1000XT GPS, Qstarz)	Food environment data source: lists of food store places from a commercial company (Reference USA); Food environment assessed: Density of takeaway food outlets and all food outlets categorized within tertiles with the highest tertile indicating more exposure to food outlets; GPS-based food exposure measures: 100 m GPS route buffer	7 days Every 60 s	Not reported	Height and weight were measured by trained research staff (using a Seca 124 Portable stadiometer and a Tanita BWB-800 portable scale, respectively), and age-specific BMI z-scores calculated relative to growth charts from the US CDC
Tamura (2018)^m^	USA	Adults	51.9 18 + years	102	GPS device (Qstarz BT-Q1000XT GPS, Qstarz)	Food environment data source: lists of food store places from the government; Food environment assessed: Density of fast-food restaurants, wait-service restaurants, corner stores, grocery stores and supermarkets; GPS-based food exposure measures: 200 m and 400 m daily mobility path on GPS points	7 days Every 30 s	Not reported	Tanita 351 scale was used to measure participants' heights and weights, wich were then used to compute BMI. Blood pressure was measured for 15–40 s SBP and DBP (based on mmHg) was assess with a Welch Allyn Vital Signs 300 monitor
Wang (2018)^n^	USA	Adults	60.8 18–65 + years	46	GPS device	Food environment data source: lists of food store places from the government; Food environment assessed: environmental context exposure index and density of fast-food restaurants, convenience stores, meat markets, pizzerias, bakeries, and candy and nut stores; GPS-based food exposure measures: 100 m 3-D GPS trajectory buffers (geolocation and time considered), 100 m GPS trajectory buffers, standard deviation ellipses with one or two standard deviations on GPS points, and minimum convex polysons of GPS points	3 weeks Every minute	Opening hours of food stores were considered	BMI was calculated by dividing the subject's weight (kg) with height in meters squared (m2) (not reported whether it was self-reported or not)

(a) Methods of exposure data collection, such as GPS trackers or mobile devices

(b) Assessment of food environment based on GPS data such as GPS points buffered or food outlets along each trip

(c) Tracking duration, such as GPS sampling frequency

(d) Temporal aspects of human mobility or food environment (e.g., opening hours)

(e) continuous or dichotomous type of diet or cardiometabolic related health outcome

All included study designs were cross-sectional

Temporal aspects were only reported in two articles, one accounting for opening hours of food stores^n^ and one using a time weighted exposure calculated based on the proportion of time individuals spent at different activity locations in the total time spent in all activity space^j^. Exposure measurement varied, most studies used route-based buffers where the buffer sizes ranged between 50 and 1500 meters^c,d−n^. Each study used different outcome measurements, except for three studies reporting on BMI^l−n^. Other outcomes were: counts of food purchases^a^, categorical food purchases^b^, junk food purchases^c^, availability, price and quality of food items^g^, specific food items based on self-reported Food Frequency Questionnaire (FFQ)^e^, self-reported fast food intake^j^, food intake collected by food reports and ecological momentary assessments^f^, snack food items and sweetened beverages^h^, diet quality index^d^, portion size^i^, specific food items and weight status^k^, and systolic and diastolic blood pressure^m^.

Out of the 14 included studies, only six studies^b,c,f,j,m,n^ consistently found associations between the food environment and diet-related or cardiometabolic outcomes. The remaining studies^a,d,e,g,h,i,k,l^ only found one or none of the several associations tested.

### Food outlet exposure and food purchases

Three studies investigated associations between food outlets and food purchases^a−c^ (Table 3**)**. Widener et al. explored associations between activity space-based access to several food outlets and grocery store purchases, convenience store purchases, and restaurant purchases, but only found that higher exposure to limited-service restaurants was associated with lower grocery store purchases and higher exposure to convenience stores was associated with higher grocery store purchases^a^. Wray et al. found associations between frequent exposure to grocery stores and advertisements and being more likely to make a restaurant purchase^b^. Sadler et al. found that being more exposed to junk food outlets was associated with higher odds of junk food purchases^c^.

**Table 3:** Results of included studies

First author (year)	Food environment exposure	Outcome	Results
Food outlets and food purchases			
Widener (2018)^a^	Grocery store count with a time weighted KD estimate activity space of 1%	Grocery store purchases	RR: 1.00; 90%CI 0.99, 1.02
		Convenience store purchases	RR: 0.99; 90%CI 0.96, 1.03
		Restaurant purchases	RR: 0.99; 90%CI 0.99, 1.01
		Purchases for now	RR: 0.99; 90%CI 0.99, 1.00
		Purchases for later	RR: 1.01; 90%CI 1.00, 1.02
	Limited-service restaurant count with a time weighted KD estimate activity space of 1%	Grocery store purchases	**RR: 0.99; 90%CI 0.99, 0.99**
		Convenience store purchases	RR: 1.00; 90%CI 0.99, 1.02
		Restaurant purchases	RR: 1.01; 90%CI 1.00, 1.01
		Purchases for now	**RR: 1.01; 90%CI 1.00, 1.01**
		Purchases for later	**RR: 0.99; 90%CI 0.99, 0.99**
	Convenience store count with a time weighted KD estimate activity space of 1%	Grocery store purchases	**RR: 1.02; 90%CI 1.00, 1.03**
		Convenience store purchases	RR: 1.00; 90%CI 0.96, 1.04
		Restaurant purchases	RR: 1.01; 90%CI 0.97, 1.05
		Purchases for now	RR: 0.99; 90%CI 0.98, 1.00
		Purchases for later	RR: 1.01; 90%CI 0.99, 1.02
	Fruit and vegetable count with a time weighted KD estimate activity space 1%	Grocery store purchases	RR: 0.99; 90%CI 0.95, 1.04
		Convenience store purchases	RR: 0.95; 90%CI 0.81, 1.09
		Restaurant purchases	RR: 1.01; 90%CI 0.97, 1.05
		Purchases for now	RR: 1.01; 90%CI 0.97, 1.04
		Purchases for later	RR: 0.98; 90%CI 0.95, 1.02
	Grocery store count with a time weighted KD estimate activity space of 10%	Grocery store purchases	RR: 1.00; 90%CI 0.98, 1.03
		Convenience store purchases	RR: 1.06; 90%CI 0.99, 1.15
		Restaurant purchases	RR: 1.01; 90%CI 0.99, 1.02
		Purchases for now	RR: 1.01; 90%CI 0.99, 1.03
		Purchases for later	RR: 1.01; 90%CI 0.99, 1.03
	Convenience store count with a time weighted KD estimate activity space of 10%	Grocery store purchases	RR: 0.99; 90%CI 0.98, 1.01
		Convenience store purchases	RR: 0.99; 90%CI 0.96, 1.02
		Restaurant purchases	RR: 1.00; 90%CI 0.99, 1.08
		Purchases for now	RR: 1.00; 90%CI 0.99, 1.01
		Purchases for later	RR: 0.99; 90%CI 0.99, 1.01
	Limited-service restaurant count with a time weighted KD estimate activity space 10%	Grocery store purchases	RR: 1.01; 90%CI 0.99, 1.03
		Convenience store purchases	RR: 1.01; 90%CI 0.95, 1.07
		Restaurant purchases	RR: 0.99; 90%CI 0.98, 1.01
		Purchases for now	RR: 0.99; 90%CI 0.98, 1.01
		Purchases for later	RR: 1.01; 90%CI 0.99, 1.02
	Fruit and vegetable count with a time weighted KD estimate activity space of 1%	Grocery store purchases	RR: 1.02; 90%CI 0.95, 1.10
		Convenience store purchases	RR: 0.78; 90%CI 0.56, 1.02
		Restaurant purchases	RR: 1.03; 90%CI 0.96, 1.10
		Purchases for now	RR: 1.00; 90%CI 0.94 1.06
		Purchases for later	RR: 0.99; 90%CI 0.99, 1.06
Wray (2021)^b^	Frequent exposure to quick service restaurants and ads	Quick service purchases	OR: 1.50; 95%CI 0.89, 2.60
		Restaurant purchases	OR: 0.88; 95%CI 0.47, 1.66
		Grocery purchases	OR: 0.83; 95%CI 0.55, 1.27
		Variety purchases	OR: 1.33; 95%CI 0.70, 2.57
		All types of purchases	OR: 1.01; 95%CI 0.68, 1.51
	Frequent exposure to restaurants and ads	Quick service purchases	OR: 0.90; 95%CI 0.43, 1.90
		Restaurant purchases	OR: 0.35; 95%CI 0.09, 1.12
		Grocery purchases	OR: 0.98; 95%CI 0.49, 1.93
		Variety purchases	OR: 0.51; 95%CI 0.15, 1.56
		All types of purchases	OR: 0.94; 95%CI 0.51, 1.82
	Frequent exposure to grocery stores and ads	Quick service purchases	OR: 2.72; 95%CI 0.62, 15.3
		Restaurant purchases	**OR: 6.79; 95%CI 1.06, 44.9**
		Grocery purchases	OR: 1.78; 95%CI 0.44, 7.55
		Variety purchases	OR: 4.66; 95%CI 0.46, 82.8
		All types of purchases	OR: 2.88; 95%CI 0.75, 14.9
	Frequent exposure to variety outlets and ads	Quick service purchases	OR: 0.22, 95%CI 0.05, 0.83
		Restaurant purchases	OR: 1.25; 95%CI 0.27, 5.08
		Grocery purchases	OR: 0.88; 95%CI 0.33, 2.31
		Variety purchases	OR: 0.87; 95%CI 0.17, 4.35
		All types of purchases	OR: 0.56; 95%CI 0.22, 1.48
Sadler (2016)^**c**^	Junk food outlets	Junk food purchase	**OR: 1.17; 95%CI 1.14, 1.21**
	1-min increase in exposure to junk food outlets	Junk food purchase	**OR: 1.13; 95%CI 1.06, 1.20**
	Junk food outlet exposure through trips made by car	Junk food purchase	**OR: 1.22; 95%CI 1.16, 1.28**
	Junk food outlet exposure through trip made by bus	Junk food purchase	OR: 1.02; 95%CI 0.92, 1.13
	Junk food exposure through trips made to school	Junk food purchase	**OR: 1.22; 95%CI 1.12, 1.33**
	Junk food exposure through trips made from school	Junk food purchase	**OR: 1.12; 95%CI 1.08, 1.16**
	1-min junk food exposure among women	Junk food purchase	**OR: 1.19; 95%CI 1.15, 1.24**
	1-min junk food exposure among men	Junk food purchase	**OR: 1.12; 95%CI 1.06, 1.19**
Food outlets and dietary intake			
Shearer (2015)^e^	GPS based accessibility to convenience stores	Fruit and vegetable consumption	***r* = 0.14**
		Calories	*r* = − 0.02
		Diet quality	*r* = 0.14
		Frequency of fast food consumption	*r* = − 0.07
		Frequency of ready-made food consumption	*r* = 0.08
	GPS based accessibility to fast food locations	Fruit and vegetable consumption	*r* = 0.10
		Calories	*r* = − 0.03
		Diet quality	*r* = 0.09
		Frequency of fast food consumption	*r* = − 0.09
		Frequency of ready-made food consumption	*r* = 0.04
	GPS based accessibility to restaurants	Fruit and vegetable consumption	*r* = 0.07
		Calories	*r* = − 0.03
		Diet quality	*r* = 0.08
		Frequency of fast food consumption	*r* = − 0.04
		Frequency of ready-made food consumption	*r* = 0.04
	GPS based accessibility to grocery stores	Fruit and vegetable consumption	*r* = 0.10
		Calories	*r* = − 0.01
		Diet quality	*r* = 0.06
		Frequency of fast food consumption	*r* = − 0.02
		Frequency of ready-made food consumption	*r* = 0.06
Zenk (2011)^e^	1 neighborhood fast food outlet density with one standard deviation ellipse	Saturated fat intake	β 0.19; SE: 2.00
	2 + neighborhood fast food outlet density with one standard deviation ellipse	Saturated fat intake	β − 2.91; SE: 1.80
	1 neighborhood fast food outlet density with one standard deviation ellipse	Fruit and vegetable intake	β − 0.01; SE: 0.14
	2 + neighborhood fast food outlet density with one standard deviation ellipse	Fruit and vegetable intake	β − 0.07; SE: 0.13
	1 neighborhood fast food outlet density with one standard deviation ellipse	Whole grain intake	β 0.12; SE: 0.14
	2 + neighborhood fast food outlet density with one standard deviation ellipse	Whole grain intake	β: − 0.08; SE: 0.13
	Ellipse fast food outlet density	Saturated fat intake	β 1.29; SE: 1.18
		fruit and vegetable intake	β 0.07; SE: 0.08
		whole grain intake	β 0.04; SE: 0.09
	1 neighborhood fast food outlet density on the daily path area	Saturated fat intake	β 0.29; SE: 1.94
	2 + neighborhood fast food outlet density on the daily path area	Saturated fat intake	β − 2.78; SE: 1.66
	1 neighborhood fast food outlet density on the daily path area	Fruit and vegetable intake	β 0.01; SE: 0.14
	2 + neighborhood fast food outlet density on the daily path area	Fruit and vegetable intake	β − 0.03; SE: 0.12
	1 neighborhood fast food outlet density on the daily path area	Whole grain intake	β 0.13; SE: 0.14
	2 + neighborhood fast food outlet density on the daily path area	Whole grain intake	β: − 0.02; SE: 0.12
	Fast food outlet density on the daily path area	Saturated fat intake	**β 3.72; SE: 1.42**
		fruit and vegetable intake	β − 0.09; SE: 0.10
		Whole grain intake	**β **− **0.27; SE: 0.10**
	Supermarket availability with one standard deviation ellipse	Saturated fat intake	β 0.24; SE: 1.59
		Fruit and vegetable intake	β − 0.02; SE: 0.11
		Whole grain intake	β − 0.18; SE: 0.11
	Supermarket availability on the daily path area	Saturated fat intake	β 0.95; SE: 1.80
		Fruit and vegetable intake	β − 0.04; SE: 0.12
		Whole grain intake	β − 0.17; SE: 0.13
Elliston (2020)^f^	Number of food outlets	Eating/Non-eating behavior	AUC-ROC > 0.5, p < 0.001
Gustafson (2013)^g^	Availability of healthy food venues	Fruit and vegetable intake	OR: 0.91; 95%: 0.52, 1.50
		Sweetened beverages	OR: 0.66; 95%CI 0.36, 1.24
		Red meat	OR: 1.04; 95%CI 0.59, 1.83
		Milk	OR: 0.84; 95%CI 0.46, 1.57
		Baked good and sweets	OR: 0.82; 95% CI 0.47, 1.41
		Cereal	OR: 1.24; 95%CI 0.70, 2.20
Ghosh Roy (2019)^h^	Food environment around home	Snack intake	OR: 0.7; 95%CI 0.6–1.0
Seto (2016)^i^	All types of food outlets	Portion size	β 0.32; 95% 0.16, 0.49
	Bakery	Portion size	β 5.27; 95% 1.36, 9.17
	Bar	Portion size	β 6.12; 95% 2.44, 9.79
	Café	Portion size	β 6.16; 95% 3.16, 9.15
	Convenience stores	Portion size	β 1.75; 95% 0.53, 2.98
	Food	Portion size	β 0.33; 95% 0.16, 0.50
	Grocery or supermarket	Portion size	β 12.21; 95% 0.16, 0.49
	Meal delivery	Portion size	β 14.39; 95% 4.55, 24.23
	Meal takeaway	Portion size	β 14.71; 95% 7.57, 21.84
	Restaurant	Portion size	β 0.45; 95% 0.23, 0.67
Liu (2020)^j^	Fast food outlets in a 500-m buffer	Fast food consumption	**IRR: 1.08; 95% CI 0.99,1.16**
	Fast food outlets in a 1-km buffer	Fast food consumption	**IRR:1.14; 95% CI 1.02,1.26**
	Fast food outlets in a 1,5-km buffer	Fast food consumption	**IRR:1.14; 95% CI 1.00,1.29**
	Ratio of fast food outlets	Fast food consumption	**IRR: 1.48; 95% CI 1.03,2.12S**
Christian (2012)^k^	Activity based retailed food environment index	Whole grain intake	OR: 0.83; 95%CI 0.70,0.90
		Fruits and vegetable intake	OR: 0.86; 95%CI 0.72, 1.02
		Added sugar	OR: 0.93; 95%CI 0.80, 1.08
		Red meat	OR: 1.05; 95%CI 0.94, 1.18
		Fried potatoes	OR: 0.98; 95%CI 0.86, 1.11
		Overweight	OR: 1.02; 95%CI 0.91, 1.14
		Obesity	OR: 1.18; 95%CI 1.00, 1.38
Food intake and metabolic outcomes			
Burgoine (2015)^l^	All food outlets around home and school in the second tertile	Body Mass Index	β: 0.16; 95%CI − 0.44, 0.75
	All food outlets around home and school in the third tertile	Body Mass Index	β: − 0.15; 95%CI − 0.76, 0.45
	Takeaway food outlets around home and school in the second tertile	Body Mass Index	β: 0.32; 95%CI − 0.29, 0.94
	Takeaway food outlets around home and school in the third tertile	Body Mass Index	β: 0.15; 95%CI − 0.44, 0.75
Tamura (2018)^m^	Fast food outlets within 200 m of GPS-based buffers	Body Mass Index	β: − 0.22; 95%CI − 0.47, 0.03
		Systolic blood pressure	**β: **− **0.57; 95%CI **− **1.08, -0.06**
		Diastolic blood pressure	**β: **− **0.36; 95%CI -0.70, -0.02**
	Wait service restaurants within 200 m of GPS-based buffers	Body Mass Index	β: − 0.19; 95%CI − 0.43, 0.06
		Systolic blood pressure	**β: **− **0.53; 95%CI **− **0.98, -0.09**
		Diastolic blood pressure	**β: **− **0.30; 95%CI **− **0.60, -0.00**
	Corner stores within 200 m of GPS-based buffers	Body Mass Index	β: − 0.52; 95%CI − 2.62, 1.58
		Systolic blood pressure	**β: **− **3.04; 95%CI **− **5.81, -0.28**
		Diastolic blood pressure	β: − 1.53; 95%CI − 3.60, 0.53
	Grocery stores within 200 m of GPS-based buffers	Body Mass Index	β: − 0.27; 95%CI − 0.59, 0.06
		Systolic blood pressure	**β: **− **1.11; 95%CI **− **1.88, **− **0.34**
		Diastolic blood pressure	**β: **− **0.68; 95%CI **− **1.28, **− **0.08**
	Supermarkets within 200 m of GPS-based buffers	Body Mass Index	β: − 0.67; 95%CI − 1.36, 0.01
		Systolic blood pressure	**β: **− **1.69; 95%CI **− **3.40, 0.02**
		Diastolic blood pressure	β: − 1.14; 95%CI − 2.33, 0.06
	Fast food outlets within 400 m of GPS-based buffers	Body Mass Index	β: − 0.22; 95%CI − 0.50, 0.05
		Systolic blood pressure	**β: -0.60; 95%CI **− **1.16, − 0.05**
		Diastolic blood pressure	β: − 0.35; 95%CI − 0.70, 0.00
	Wait service restaurants within 400 m of GPS-based buffers	Body Mass Index	β: − 0.19; 95%CI − 0.49, 0.11
		Systolic blood pressure	**β: **− **0.63; 95%CI **− **1.13, − 0.13**
		Diastolic blood pressure	β: − 0.35; 95%CI − 0.71, 0.00
	Corner stores within 400 m of GPS-based buffers	Body Mass Index	β: − 1.02; 95%CI − 3.48, 1.45
		Systolic blood pressure	β: − 3.30; 95%CI − 6.77, 0.16
		Diastolic blood pressure	β: − 1.15; 95%CI − 3.74, 1.45
	Grocery stores within 400 m of GPS-based buffers	Body Mass Index	β: − 0.37; 95%CI − 0.82, 0.08
		Systolic blood pressure	**β: **− **1.34; 95%CI **− **2.34, − 0.33**
		Diastolic blood pressure	β: − 0.82; 95%CI − 1.70, 0.06
	Supermarkets within 400 m of GPS-based buffers	Body Mass Index	**β: **− **0.93; 95%CI **− **1.76, **− **0.10**
		Systolic blood pressure	**β: **− **2.50; 95%CI **− **4.85, − 0.16**
		Diastolic blood pressure	**β: − 1.57; 95%CI − 3.01, -0.13**
Wang (2018)^n^	Unhealthy food environment exposure index based on environmental context cubes with inverse-square distance decay function in various spatial and temporal resolutions	Body Mass Index	**OR: 6.81; 95%CI 1.76, 45.3**
	Unhealthy food environment exposure index based on environmental context cubes with negative-exponent distance decay function in different spatial and temporal resolutions	Body Mass Index	**OR: 3.13; 95%CI 1.08, 11.47**

Results are presented in risk ratios (RR) or odds ratios (OR) if outcomes are categorical or binary and beta coefficients (β) if outcomes are continuous. Confidence intervals (CI) are shown in either 90% or 95%. Other results are presented as Pearson correlation coefficients (*r*), area under the curve for the receiver operating characteristic curve (AUC-ROC), kernel densities (KD), and incidence rate ratios (IRR). Effect estimates in bold font indicate a statistical significance (P < 0.05)

### Food outlet exposure and dietary intake

Eight studies^d,e,f,g,h,i,j,k^ investigated associations between food outlets and dietary intake, of which two studies consistently found significant associations^f,j^. For instance, Liu et al. found that greater exposure to fast food outlets was associated with higher fast food consumption^j^. Four studies showed inconsistent effect estimates that did not indicate for example whether greater exposure to healthy food outlets was associated with higher intake of healthy food products such as fruit and vegetables^g,h,i,k^. The remaining studies reported that only one of several tested associations was statistically significant^d,e^. For instance, Zenk et al. explored the association of activity space-based exposure to convenience stores, fast food locations, restaurants, or grocery stores separately with saturated fat intake, fruit and vegetable intake, and whole grain intake, but only found that greater fast food outlet density on the daily path area was associated with a higher saturated fat intake and lower whole grain intake^e^ **(**Table 3**).**

### Food outlet exposure and metabolic outcomes

Three studies^l,m,n^ investigated the food environment in relation to metabolic outcomes in which two studies found that higher exposure to an unhealthy food environment was associated with higher odds of being overweight^n^ and higher exposure to supermarkets was associated with a lower BMI^m^. This association was not found by Burgoine et al. where authors investigated associations between food outlets and takeaway food outlets around the home or school environment and BMI^l^. Tamura et al. also found that within 200 m of GPS-based buffers more exposure to corner stores, grocery stores and even fast food outlets and waited service restaurants, was associated with lower systolic blood pressure and diastolic pressure. When authors of this study measured GPS-based buffers within 400 m associations were only found between more exposure to supermarkets and lower diastolic blood pressure^m^ **(**Table 3**).**

## Discussion

In this systematic review we summarized the evidence regarding the association between GPS-based food environment exposures and diet-related and cardiometabolic health outcomes. Based on a small and highly heterogeneous sample of studies, we found no consistent evidence for an association between GPS-based food outlet exposure and diet-related or cardiometabolic health outcomes. Most studies examined a range of determinants and outcomes with only a few of these being statistically significant or representing meaningful associations.

The methods used to derive GPS-based food environment exposures varied as reported in an extensive methodological review [38], with regard to the type of devices used to collect GPS data, duration of GPS data collection, and methods to operationalize exposures (e.g., buffer types, buffer sizes, and GPS-point-based buffers). It has been recommended that daily path areas, in particular with smaller buffer sizes, give a better estimation of activity space compared to other measures used such as the standard deviation ellipse [38]. However, our results demonstrate that there is no clear consensus about the most suitable buffer size for measuring exposure to the food environment because associations were inconsistent regardless of activity space operationalization. Even when studies use smaller buffer sizes (e.g., 50 m) of daily path areas no meaningful or consistent results were found [39, 40].

We purposefully included studies with different diet-related and cardiometabolic health outcomes to establish whether associations with more proximal outcomes (e.g., food purchases) would be more consistent than associations with more distal outcomes (e.g., blood pressure). However, this was not the case, and we found the same level of inconsistency as in reviews on the food environment-food intake-health association using place-based exposures [10, 41–43].

Next to uncertainties about the measurement of exposure to the food environment, it has been suggested that the operationalization of the outcome measurement could also cause inconsistent results [42]. Self-reported dietary intake data is prone to recall bias, so other methods that require less recall by participants, such as momentary assessment tools, could be considered for future studies [32, 42]. Such tools can also aid in determining whether the use of food retailers within the area participants are exposed to are explaining any observed exposure – intake association.

Even though one of the potential advantages of measuring GPS-based exposure to the food environment is that the duration of exposure can be taken into account [44], only two studies included in our review considered temporal aspects [45, 46]. It may also be of interest to consider selective daily mobility bias (SDMB) which arises when individuals’ exposure is partly due to their choice to go to this location [47]. One of the explanations for individuals’ choice to go to a location is because they were visiting that area and were therefore exposed to the food environment. Another explanation could be that individuals intentionally visited a certain area to purposely visit a specific food outlet or took a certain route to that food environment. In our review, two studies mentioned the SDMB. Burgoine et al. [48] directly tested one of the implications from Chaix et al. [30] who suggested addressing SDMB by comparing GPS actual route exposures with modelled GIS route exposures. However, Burgoine et al. found no evidence for the potential impact of SDM, as modelled GIS and actual GPS routes generated similar associations with BMI. One of the reasons was that the authors were unable to study route choice based on preferences related to BMI. This study sample also included children only, authors therefore suggested to replicate this study with adults in which exposure in wider activity spaces can be measured. Widener et al. [49] considered individuals’ food retailers preferences and found that household food shoppers purchased more food items at grocery stores and have a higher number of grocery stores in their food environment.

### Strengths and limitations

Strengths of this review comprised an extensive search in seven databases conducted by a medical information specialist where several types of food environments and a variety of diet-related and cardiometabolic health outcomes were considered. Others strengths are that two independent researchers screened articles and the use of a validated quality assessment tool [36] to perform the quality assessment of the included articles. Limitations include the fact that most studies were conducted in the USA or Canada and the results of the study may therefore not be generalizable to other populations in other countries. We were only able to include a small number of studies resulting in heterogeneity which made it impossible to perform a meta-analysis. Although we included all ages in our review, most of the studies were conducted among younger adults of which the results may not be applicable to other generations: older adults and children may have smaller activity space than adults. Additionally, parents may have more influence on their dietary choices compared to (young) adults. Other limitations are the relatively small sample sizes in the included studies, which may have limited the generalizability of the results. Also, the tracking days of GPS may not have fully captured individuals’ exposure, but only for that timing of measurement. Finally, studies did not report information on representativeness of the samples, non-respondents, or sample size calculations, increasing the risk of bias.

### Research agenda

To advance our understanding of how to use GPS tracking data for food environment research, there are at least six conceptual and methodological challenges to overcome.

First, various uncertainties emerge due to inconsistencies in GPS data processing. This includes, for example, generating activity spaces based on all GPS points, so both locations and routes between locations, while other studies only base food retailer exposures on GPS-derived locations. Such processing decisions likely significantly affect the results [27] because generating activity spaces based on all GPS points captures all potential food outlets within that activity space. Using retailer-based exposures only can result in potential underestimations of exposure [27].

Second, while buffer analysis remains the gold standard for assessing people’s health-influencing geographic context [50], there is significant heterogeneity in the buffer sizes and types were adopted in GPS-based food studies. To provide guidance on the best approach to operationalize GPS-based food environment exposures, studies should 1) investigate the interrelation between activity spaces and exposures (e.g. using bodycams, to determine how much extra (relevant) exposure is captured when all GPS-points are included), and 2) investigate the direct and indirect behavioral pathways through which individuals respond to food retailer exposure to inform buffer sizes along GPS-routes.

Third, and closely related, the methodological variations in the processing algorithms warrant cautionary comparisons between studies. GPS studies conducted across different (sub)disciplines may report study methods and/or results in different levels of detail. For example, some studies reported the GPS sampling frequency [39, 40], whereas others did not [49, 51]. Some studies also noted the potential loss of GPS signal in certain areas and have taken measures to account for or acknowledge this limitation [24, 29, 40, 45, 48], while the rest of the studies did not report this. Consequently, the quality assessment of GPS studies could be biased. As is common for various aspects in health research [52], we suggest defining a reporting standard that indicates key items to improve comparability across GPS studies.

Fourth, with only a few exceptions [46, 49], studies that typically use all collected GPS points can reduce the risk of exposure. This is because using GPS points captures food outlets within individuals’ activity space more comprehensively compared to studies that only use GPS-derived activity locations, which may overlook the travel between these activity locations [27]. Therefore, assessing exposure based on all GPS points to explore exposure-outcome associations can mitigate the risk of exposure misclassification.

Fifth, the existence of SDMB might influence the methods for generating activity space-based exposure assessment (e.g., whether using the actual path or the shortest path between activity locations) [23, 48, 53]. Consequently, we encourage future studies to look into the existence of SDMB by comparing results for actual and shortest path and the development of other conceptual and analytical approaches to overcome this potential bias.

Sixth, in addition to time-weighted exposure [45], methodological rigor in terms of refined exposure assessments can also be achieved by accounting for time as conceptually suggested in Time Geography^(54)^, either through taking people's time constraints and store opening hours into account. Moreover, analyzing food exposure from a time series perspective (e.g., cumulative exposures) may also give a better understanding of the causality between the food environment and health outcomes.

Given that using GPS-based food environment exposures as compared to static exposures does not seem to generate more consistent associations with behavioral and health outcomes, the main value of future GPS studies may lie in their contribution to better operationalizing activity spaces and understanding human behaviors in relation to momentary and cumulative exposures. Once more precise food environment exposures can be operationalized, larger-scale longitudinal or (quasi) experimental studies can help unravel causal exposure-outcome relations.

## Conclusions

This systematic review provided an update on the state of evidence on GPS-based measured food environment and diet-related and cardiometabolic health outcomes. Although many studies advocate the use of GPS-based methods, the current but limited evidence base does not provide strong evidence for consistent associations with diet-related and cardiometabolic health outcomes.

### Supplementary Information

**Additional file 1: Table S1a.** History and Search Details Pubmed October 31, 2022.** Table S1b.** History and Search Details Embase October 31, 2022. **Table S1c.** History and Search Details Cinahl (Ebsco) October 31, 2022.** Table S1d.** History and Search Details PsycInfo (Ebsco) October 31, 2022. **Table 1e.** History and Search Details WEB OF SCIENCE Core Collection October 31, 2022. **Table S1f**. History and Search Details SCOPUS October 31, 2022. **Table S1g.** History and Search Details IBSS October 31, 2022. **Table S2.** Data extraction table. **Table S3.** Quality assessment according to the Newcastle-Ottawa Scale (NOS) and included items from Cetateanu et al.

## Data Availability

No datasets were generated or analyzed during this study.
