# Supplementary material for: Global positioning system-based food environment exposures, diet-related, and cardiometabolic health outcomes: a systematic review and research agenda
Source: Int J Health Geogr. 2024 Feb 6;23:3. doi: 10.1186/s12942-024-00362-x (PMC10848400; doi:10.1186/s12942-024-00362-x)
Supplement: Supplementary file 1 — Additional file 1: Table S1a. History and Search Details Pubmed October 31, 2022. Table S1b. History and Search Details Embase October 31, 2022. Table S1c. History and Search Details Cinahl (Ebsco) October 31, 2022. Table S1d. History and Search Details PsycInfo (Ebsco) October 31, 2022. Table 1e. History and Search Details WEB OF SCIENCE Core Collection October 31, 2022. Table S1f. History and Search Details SCOPUS October 31, 2022. Table S1g. History and Search Details IBSS October 31, 2022. Table S2. Data extraction table. Table S3. Quality assessment according to the Newcastle-Ottawa Scale (NOS) and included items from Cetateanu et al. [file 12942_2024_362_MOESM1_ESM.docx]

**Additional files**

## **Additional file 1:Table S1a.** History and Search Details Pubmed October 31, 2022

| **Set** | **PubMed Query** | **Results** |
| --- | --- | --- |
| #4 | **#1 AND #2 AND #3** | 1,097 |
| #3 | "Diet"[Mesh] OR "Food Quality"[Mesh] OR "Energy Intake"[Mesh] OR (food[tiab] AND (quality[tiab] OR purchas*[tiab] OR consumption[tiab] OR consuming[tiab] OR intake[tiab])) OR (( energy[tiab] OR nutrient*[tiab] OR habitual[tiab] OR diet[tiab] OR dietary[tiab] OR kcal[tiab] OR kilocal*[tiab] OR calories[tiab] OR calory[tiab] OR caloric[tiab] OR food[tiab]) AND intake[tiab]) OR “caloric restriction*”[tiab] OR (diet*[tiab] AND (quality[tiab] OR intake[tiab])) OR "Metabolic Syndrome"[Mesh] OR "Cardiovascular Diseases"[Mesh] OR "Heart Disease Risk Factors"[Mesh] OR "Glucose Intolerance"[Mesh] OR "Blood Glucose"[Mesh] OR "Hyperglycemia"[Mesh] OR "Glycated Hemoglobin A"[Mesh] OR "Diabetes Mellitus"[Mesh:NoExp] OR "Diabetes Mellitus, Type 2"[Mesh] OR "Prediabetic State"[Mesh] OR "Insulin Resistance"[Mesh] OR "Hyperinsulinism"[Mesh] OR "Blood Pressure"[Mesh] OR "Hypertension"[Mesh] OR "Arterial Pressure"[Mesh] OR "Triglycerides"[Mesh] OR "Cholesterol"[Mesh] OR "Lipoproteins, HDL"[Mesh] OR "Lipoproteins, LDL"[Mesh] OR "Lipoproteins, VLDL"[Mesh] OR "Lipoproteins, IDL"[Mesh] OR "Obesity, Abdominal"[Mesh] OR "Body Weight Changes"[Mesh] OR "Body Weights and Measures"[Mesh] OR "Dyslipidemias"[Mesh] OR "Waist Circumference"[Mesh] OR "Waist-Hip Ratio"[Mesh] OR "metabolic syndrome"[tiab] OR “cardiovascular risk*”[tiab] OR “cardiovascular dis*”[tiab] OR cardiometabolic[tiab] OR "glucose intoleran*"[tiab] OR "glucose toleran*"[tiab] OR "glucose dysregulation"[tiab] OR "igt"[tiab] OR "blood glucose"[tiab] OR "blood sugar"[tiab] OR "plasma glucose"[tiab] OR "glucose level*"[tiab] OR "glucose blood"[tiab] OR "fasting glucose"[tiab] OR "hyperglycem*"[tiab] OR "hyperglycaem*"[tiab] OR "Hb A1c"[tiab] OR "HbA1c"[tiab] OR "Hemoglobin A1c"[tiab] OR "diabet*"[tiab] OR "type 2 dm"[tiab] OR "type II dm"[tiab] OR "dm type 2"[tiab] OR "dm type II"[tiab] OR "NIDDM"[tiab] OR "IDDM"[tiab] OR "insulin resistan*"[tiab] OR "insulin sensitiv*"[tiab] OR "insulin insensitiv*"[tiab] OR "homa-ir"[tiab] OR "homa2"[tiab] OR "hyperinsulin*"[tiab] OR "prediabet*"[tiab] OR "blood pressure"[tiab] OR "diastolic pressure"[tiab] OR "systolic pressure"[tiab] OR "pulse pressure"[tiab] OR "arterial pressure"[tiab] OR "aortic pressure"[tiab] OR "hypertensi*"[tiab] OR "triglyceride*"[tiab] OR "triacylglycerol*"[tiab] OR "triacetylglycerol"[tiab] OR "triacetyl-glycerol"[tiab] OR "trielaidin"[tiab] OR "trioleoylglycerol"[tiab] OR "trioleyl glycerol"[tiab] OR "glycerol trioleate"[tiab] OR "trioleate-glycerin"[tiab] OR "hypertriglyceridemi*"[tiab] OR "cholesterol"[tiab] OR "hdl"[tiab] OR "ldl"[tiab] OR "vldl"[tiab] OR "idl"[tiab] OR "density lipoprotein*"[tiab] OR "hdl lipoprotein*"[tiab] OR "low density lipoprotein*"[tiab] OR "high density lipoprotein*"[tiab] OR "very low lipoprotein*"[tiab] OR "obesit*"[tiab] OR "obese*"[tiab] OR "body mass index"[tiab] OR "bmi"[tiab] OR "body weight"[tiab] OR "body fat*"[tiab] OR "adiposit*"[tiab] OR "waist hip ratio*"[tiab] OR "waist to hip*"[tiab] OR "waist hip ratio*"[tiab] OR "waist circumference*"[tiab] OR "weight change*"[tiab] OR "weight gain*"[tiab] OR "weight loss*"[tiab] OR "weight reduction*"[tiab] OR "dyslipem*"[tiab] OR "dyslipidem*"[tiab] OR "dyslipaem*"[tiab] OR "dyslipidaem*"[tiab] OR "dyslipoprotein*"[tiab] OR "hyperlipemi*"[tiab] OR "hyperlipid*"[tiab] OR "lipidemi*"[tiab] OR "lipidaemi*"[tiab] OR "lipemi*"[tiab] OR "hypercholesterolemi*"[tiab] OR "hypercholesteremi*"[tiab] OR "hyperlipoproteinemi*"[tiab] OR "hypoprebetalipoproteinemi*"[tiab] | 5,183,193 |
| #2 | "Geographic Information Systems"[Mesh] OR "Smartphone"[Mesh] OR "Ecological Momentary Assessment"[Mesh] OR "Spatial Analysis"[Mesh] OR “geographic information system*”[tiab] OR “geographical information system*”[tiab] OR “Global Positioning System*”[tiab] OR GPS[tiab] OR (tracker*[tiab] AND (route*[tiab] OR location[tiab] OR address[tiab])) OR “mobile phone*”[tiab] OR “mobile telephone*”[tiab] OR smartphone*[tiab] OR “smart phone*”[tiab] OR “Ecological Momentary Assessment*”[tiab] OR EMA[tiab] OR “Geographical momentary assessment*”[tiab] OR “activity spac*”[tiab] OR “spatial epidemiology”[tiab] | 101,678 |
| #1 | "Food Supply"[Mesh] OR "Fast Foods"[Mesh] OR (food[tiab] AND (environment*[tiab] OR suppl*[tiab] OR outlet*[tiab] OR expos*[tiab] OR desert*[tiab] OR swamp*[tiab] OR availab*[tiab] OR access*[tiab] OR proximit*[tiab] OR densit*[tiab] OR store*[tiab] OR shop*[tiab])) OR foodscape[tiab] OR restaurant*[tiab] OR fastfood[tiab] OR fast-food[tiab] OR supermarket*[tiab] OR retail[tiab] | 229,427 |

## **Additional file 1: Table S1b.** History and Search Details Embase October 31, 2022

| **Set** | **Embase.com Query** | **Results** |
| --- | --- | --- |
| #5 | #4 NOT ('conference abstract'/it OR 'conference review'/it) | 1,083 |
| #4 | #1 AND #2 AND #3 | 1,477 |
| #3 | 'diet'/exp OR 'food quality'/exp OR 'caloric intake'/exp OR 'nutrient intake'/exp OR 'metabolic syndrome X'/exp OR 'cardiovascular disease'/exp OR 'cardiovascular risk factor'/exp OR 'glucose intolerance'/exp OR 'glucose blood level'/exp OR 'hyperglycemia'/exp OR 'glycosylated hemoglobin'/exp OR 'diabetes mellitus'/de OR 'insulin dependent diabetes mellitus'/exp OR 'non insulin dependent diabetes mellitus'/exp OR 'impaired glucose tolerance'/exp OR 'insulin resistance'/exp OR 'hyperinsulinism'/exp OR 'blood pressure'/exp OR 'arterial pressure'/exp OR 'hypertension'/exp OR 'triacylglycerol'/exp OR 'cholesterol'/exp OR 'high density lipoprotein'/exp OR 'low density lipoprotein'/exp OR 'very low density lipoprotein'/exp OR 'intermediate density lipoprotein'/exp OR 'abdominal obesity'/exp OR 'body weight change'/exp OR 'body weight'/exp OR 'dyslipidemia'/exp OR 'waist circumference'/exp OR 'waist hip ratio'/exp OR ((food AND (quality OR purchas* OR consumption OR consuming OR intake)) OR ((energy OR nutrient* OR habitual OR diet OR dietary OR kcal OR kilocal* OR calories OR calory OR caloric OR food) AND (intake)) OR ‘caloric restriction*’ OR (diet* AND (quality OR intake)) OR ‘metabolic syndrome’ OR ‘cardiovascular risk*’ OR ‘cardiovascular dis*’ OR cardiometabolic OR ‘glucose intoleran*’ OR ‘glucose toleran*’ OR ‘glucose dysregulation’ OR igt OR ‘blood glucose’ OR ‘blood sugar’ OR ‘plasma glucose’ OR ‘glucose level*’ OR ‘glucose blood’ OR ‘fasting glucose’ OR hyperglycem* OR hyperglycaem* OR ‘Hb A1c’ OR ‘HbA1c’ OR ‘Hemoglobin A1c’ OR diabet* OR ‘type 2 dm’ OR ‘type II dm’ OR NIDDM OR IDDM OR ‘insulin resistan*’ OR ‘insulin sensitiv*’ OR ‘homa-ir’ OR homa2 OR hyperinsulin* OR prediabet* OR ‘blood pressure’ OR ‘diastolic pressure’ OR ‘systolic pressure’ OR ‘pulse pressure’ OR ‘arterial pressure’ OR ‘aortic pressure’ OR hypertensi* OR triglyceride* OR triacylglycerol* OR triacetylglycerol OR ‘triacetyl-glycerol’ OR trielaidin OR trioleoylglycerol OR ‘trioleyl glycerol’ OR ‘glycerol trioleate’ OR ‘trioleate-glycerin’ OR hypertriglyceridemi* OR cholesterol OR hdl OR vdl OR ldl OR idl OR ‘high density lipoprotein*’ OR ‘hdl lipoprotein*’ OR ‘low-density lipoprotein*’ OR ‘very low lipoprotein*’ OR obesit* OR obese* OR ‘body mass index’ OR bmi OR ‘body weight’ OR ‘body fat*’ OR adiposit* OR ‘waist-hip ratio*’ OR ‘waist to hip*’ OR ‘waist circumference*’ OR ‘weight change*’ OR ‘weight gain*’ OR ‘weight loss*’ OR ‘weight reduction*’ OR dyslipem* OR dyslipidem* OR dyslipaem* OR dyslipidaem* OR dyslipoprotein* OR hyperlipemi* OR hyperlipid* OR lipidemi* OR lipidaemi* OR lipemi* OR hypercholesterolemi* OR hypercholesteremi* OR hyperlipoproteinemi* OR hypoprebetalipoproteinemi*):ab,ti,kw | 8,093,804 |
| #2 | 'geographic information system'/exp OR 'smartphone'/exp OR 'ecological momentary assessment'/exp OR 'spatial analysis'/exp OR (‘geographic* information system*’ OR ‘Global Positioning System*’ OR GPS OR (tracker* AND (route* OR location OR address)) OR ‘mobile phone*’ OR ‘mobile telephone*’ OR smartphone* OR ‘smart phone*’ OR ‘Ecological Momentary Assessment*’ OR EMA OR ‘Geographical momentary assessment*’ OR ‘activity spac*’ OR ‘spatial epidemiology’):ab,ti,kw | 147,720 |
| #1 | 'catering service'/exp OR 'food desert'/exp OR 'restaurant'/exp OR 'grocery store'/exp OR 'food desert'/exp OR ((food AND (environment* OR suppl* OR outlet* OR expos* OR desert* OR swamp* OR availab* OR access* OR proximit* OR densit* OR store* OR shop*)) OR foodscape OR restaurant* OR fastfood OR fast-food OR supermarket* OR retail):ab,ti,kw | 280,324 |

## **Additional file 1: Table S1c.** History and Search Details Cinahl (Ebsco) October 31, 2022

| **Set** | **Cinahl (Ebsco) Query** | **Results** |
| --- | --- | --- |
| S4 | S1 AND S2 AND S3 | 399 |
| S3 | MH ("Nutrient Density" OR "Energy Intake" OR "Energy Density" OR "Diet" OR "Food Intake+" OR "Metabolic Syndrome X+" OR "Cardiovascular Diseases" OR "Heart Diseases+" OR "Arrhythmia" OR "Cardiac Output, Decreased" OR "Heart Arrest+" OR "Heart Failure+" OR "Heart Valve Diseases" OR "Myocardial Diseases+" OR "Coronary Disease+" OR "Myocardial Ischemia+" OR "Angina Pectoris+" OR "Coronary Occlusion+" OR "Coronary Stenosis+" OR "Pulmonary Heart Disease" OR "Diabetic Angiopathies+" OR "Embolism and Thrombosis+" OR "Hypertension+" OR "Hypotension+" OR "Ischemia+" OR "Glucose Intolerance" OR "Diabetes Mellitus+" OR "Diabetic Neuropathies+" OR "Hyperglycemia" OR "Prediabetic State" OR "Hyperinsulinism+" OR "Hemoglobin A, Glycosylated" OR "Blood Pressure+" OR "Arterial Pressure+" OR "Hypertension+" OR "Hypertension, Pulmonary" OR "Pulmonary Arterial Hypertension" OR "Triglycerides" OR "Cholesterol" OR "Cholesterol, Dietary" OR "Embolism, Cholesterol" OR "Lipoproteins, HDL Cholesterol" OR "Lipoproteins, LDL Cholesterol" OR "Lipoproteins+" OR "Obesity" OR "Obesity, Morbid" OR "Body Weight Changes" OR "Body Weight" OR "Weight Gain" OR "Weight Loss" OR "Weight Cycling" OR "Hyperlipidemia+" OR "Hypercholesterolemia+" OR "Hyperlipoproteinemia+" OR "Waist-Hip Ratio" OR "Waist Circumference") OR TI ((food AND (quality OR purchas* OR consumption OR consuming OR intake)) OR ((energy OR nutrient* OR habitual OR diet OR dietary OR kcal OR kilocal* OR calories OR calory OR caloric OR food) AND (intake)) OR “caloric restriction*” OR (diet* AND (quality OR intake)) OR “metabolic syndrome” OR “cardiovascular risk*” OR “cardiovascular dis*” OR cardiometabolic OR “glucose intoleran*” OR “glucose toleran*” OR “glucose dysregulation” OR igt OR “blood glucose” OR “blood sugar” OR “plasma glucose” OR “glucose level*” OR “glucose blood” OR “fasting glucose” OR hyperglycem* OR hyperglycaem* OR “Hb A1c” OR “HbA1c” OR “Hemoglobin A1c” OR diabet* OR “type 2 dm” OR “type II dm” OR NIDDM OR IDDM OR “insulin resistan*” OR “insulin sensitiv*” OR “homa-ir” OR homa2 OR hyperinsulin* OR prediabet* OR “blood pressure” OR “diastolic pressure” OR “systolic pressure” OR “pulse pressure” OR “arterial pressure” OR “aortic pressure” OR hypertensi* OR triglyceride* OR triacylglycerol* OR triacetylglycerol OR “triacetyl-glycerol” OR trielaidin OR trioleoylglycerol OR “trioleyl glycerol” OR “glycerol trioleate” OR “trioleate-glycerin” OR hypertriglyceridemi* OR cholesterol OR hdl OR vdl OR ldl OR idl OR “high density lipoprotein*” OR “hdl lipoprotein*” OR “low-density lipoprotein*” OR “very low lipoprotein*” OR obesit* OR obese* OR “body mass index” OR bmi OR “body weight” OR “body fat*” OR adiposit* OR “waist-hip ratio*” OR “waist to hip*” OR “waist circumference*” OR “weight change*” OR “weight gain*” OR “weight loss*” OR “weight reduction*” OR dyslipem* OR dyslipidem* OR dyslipaem* OR dyslipidaem* OR dyslipoprotein* OR hyperlipemi* OR hyperlipid* OR lipidemi* OR lipidaemi* OR lipemi* OR hypercholesterolemi* OR hypercholesteremi* OR hyperlipoproteinemi* OR hypoprebetalipoproteinemi*) OR AB ((food AND (quality OR purchas* OR consumption OR consuming OR intake)) OR ((energy OR nutrient* OR habitual OR diet OR dietary OR kcal OR kilocal* OR calories OR calory OR caloric OR food) AND (intake)) OR “caloric restriction*” OR (diet* AND (quality OR intake)) OR “metabolic syndrome” OR “cardiovascular risk*” OR “cardiovascular dis*” OR cardiometabolic OR “glucose intoleran*” OR “glucose toleran*” OR “glucose dysregulation” OR igt OR “blood glucose” OR “blood sugar” OR “plasma glucose” OR “glucose level*” OR “glucose blood” OR “fasting glucose” OR hyperglycem* OR hyperglycaem* OR “Hb A1c” OR “HbA1c” OR “Hemoglobin A1c” OR diabet* OR “type 2 dm” OR “type II dm” OR NIDDM OR IDDM OR “insulin resistan*” OR “insulin sensitiv*” OR “homa-ir” OR homa2 OR hyperinsulin* OR prediabet* OR “blood pressure” OR “diastolic pressure” OR “systolic pressure” OR “pulse pressure” OR “arterial pressure” OR “aortic pressure” OR hypertensi* OR triglyceride* OR triacylglycerol* OR triacetylglycerol OR “triacetyl-glycerol” OR trielaidin OR trioleoylglycerol OR “trioleyl glycerol” OR “glycerol trioleate” OR “trioleate-glycerin” OR hypertriglyceridemi* OR cholesterol OR hdl OR vdl OR ldl OR idl OR “high density lipoprotein*” OR “hdl lipoprotein*” OR “low-density lipoprotein*” OR “very low lipoprotein*” OR obesit* OR obese* OR “body mass index” OR bmi OR “body weight” OR “body fat*” OR adiposit* OR “waist-hip ratio*” OR “waist to hip*” OR “waist circumference*” OR “weight change*” OR “weight gain*” OR “weight loss*” OR “weight reduction*” OR dyslipem* OR dyslipidem* OR dyslipaem* OR dyslipidaem* OR dyslipoprotein* OR hyperlipemi* OR hyperlipid* OR lipidemi* OR lipidaemi* OR lipemi* OR hypercholesterolemi* OR hypercholesteremi* OR hyperlipoproteinemi* OR hypoprebetalipoproteinemi*) | 1,142,042 |
| S2 | MH "Geographic Information Systems+" OR MH "Smartphone" OR MH "Spatial Behavior" OR TI ("geographic* information system*" OR "global positioning system*" OR gps OR (tracker* AND (route* OR location OR address)) OR "mobile phone*" OR "mobile telephone*" OR smartphone* OR "smart phone*" OR "ecological momentary assessment*" OR ema OR "geographical momentary assessment*" OR "activity spac*" OR "spatial epidemiology ") OR AB ("geographic* information system*" OR "global positioning system*" OR gps OR (tracker* AND (route* OR location OR address)) OR "mobile phone*" OR "mobile telephone*" OR smartphone* OR "smart phone*" OR "ecological momentary assessment*" OR ema OR "geographical momentary assessment*" OR "activity spac*" OR "spatial epidemiology ") | 36,886 |
| S1 | MH "Restaurants" OR MH "Food Deserts" OR MH "Grocery Stores" OR TI (("food" AND ("environment*" OR "suppl*" OR "outlet*" OR "expos*" OR "desert*" OR "swamp*" OR "availab*" OR "access*" OR "proximit*" OR "densit*" OR "store*" OR "shop*")))) OR "foodscape" OR "restaurant*" OR "fastfood" OR "fast-food" OR "supermarket*" OR "retail") OR AB (("food" AND ("environment*" OR "suppl*" OR "outlet*" OR "expos*" OR "desert*" OR "swamp*" OR "availab*" OR "access*" OR "proximit*" OR "densit*" OR "store*" OR "shop*")))) OR "foodscape" OR "restaurant*" OR "fastfood" OR "fast-food" OR "supermarket*" OR "retail") | 55,689 |

## **Additional file 1: Table S1d.** History and Search Details PsycInfo (Ebsco) October 31, 2022

| **Set** | **PsycInfo (Ebsco) Query** | **Results** |
| --- | --- | --- |
| S4 | S1 AND S2 AND S3 | 206 |
| S3 | DE ("Adipocytes" OR "Aneurysms" OR "Angina Pectoris" OR "Arrhythmias (Heart)" OR "Arteriosclerosis" OR "Atherosclerosis" OR "Blood Pressure Disorders" OR "Blood Pressure" OR "Body Fat" OR "Body Mass Index" OR "Body Weight Cycling" OR "Body Weight" OR "Cardiovascular Disorders" OR "Cardiovascular Health" OR "Cerebral Arteriosclerosis" OR "Cerebral Ischemia" OR "Cerebrovascular Disorders" OR "Cholesterol" OR "Coronary Heart Disease" OR "Coronary Thromboses" OR "Diabetes Mellitus" OR "Diabetes" OR "Diastolic Pressure" OR "Embolisms" OR "Energy Expenditure" OR "Essential Hypertension" OR "Food Intake" OR "Glucose Metabolism" OR "Heart Disorders" OR "Heart Rate Variability" OR "Heart Rate" OR "Hemoglobin" OR "Hemorrhage" OR "Hyperglycemia" OR "Hypertension" OR "Hypotension" OR "Ischemia" OR "Lipid Metabolism Disorders" OR "Lipid Metabolism" OR "Lipoproteins" OR "Metabolic Syndrome" OR "Metabolism Disorders" OR "Myocardial Infarctions" OR "Obesity" OR "Overweight" OR "Syncope" OR "Systolic Pressure" OR "Thromboses" OR "Type 2 Diabetes" OR "Weight Gain" OR "Weight Loss") OR TI ((food AND (quality OR purchas* OR consumption OR consuming OR intake)) OR ((energy OR nutrient* OR habitual OR diet OR dietary OR kcal OR kilocal* OR calories OR calory OR caloric OR food) AND (intake)) OR “caloric restriction*” OR (diet* AND (quality OR intake)) OR “metabolic syndrome” OR “cardiovascular risk*” OR “cardiovascular dis*” OR cardiometabolic OR “glucose intoleran*” OR “glucose toleran*” OR “glucose dysregulation” OR igt OR “blood glucose” OR “blood sugar” OR “plasma glucose” OR “glucose level*” OR “glucose blood” OR “fasting glucose” OR hyperglycem* OR hyperglycaem* OR “Hb A1c” OR “HbA1c” OR “Hemoglobin A1c” OR diabet* OR “type 2 dm” OR “type II dm” OR NIDDM OR IDDM OR “insulin resistan*” OR “insulin sensitiv*” OR “homa-ir” OR homa2 OR hyperinsulin* OR prediabet* OR “blood pressure” OR “diastolic pressure” OR “systolic pressure” OR “pulse pressure” OR “arterial pressure” OR “aortic pressure” OR hypertensi* OR triglyceride* OR triacylglycerol* OR triacetylglycerol OR “triacetyl-glycerol” OR trielaidin OR trioleoylglycerol OR “trioleyl glycerol” OR “glycerol trioleate” OR “trioleate-glycerin” OR hypertriglyceridemi* OR cholesterol OR hdl OR vdl OR ldl OR idl OR “high density lipoprotein*” OR “hdl lipoprotein*” OR “low-density lipoprotein*” OR “very low lipoprotein*” OR obesit* OR obese* OR “body mass index” OR bmi OR “body weight” OR “body fat*” OR adiposit* OR “waist-hip ratio*” OR “waist to hip*” OR “waist circumference*” OR “weight change*” OR “weight gain*” OR “weight loss*” OR “weight reduction*” OR dyslipem* OR dyslipidem* OR dyslipaem* OR dyslipidaem* OR dyslipoprotein* OR hyperlipemi* OR hyperlipid* OR lipidemi* OR lipidaemi* OR lipemi* OR hypercholesterolemi* OR hypercholesteremi* OR hyperlipoproteinemi* OR hypoprebetalipoproteinemi*) OR AB ((food AND (quality OR purchas* OR consumption OR consuming OR intake)) OR ((energy OR nutrient* OR habitual OR diet OR dietary OR kcal OR kilocal* OR calories OR calory OR caloric OR food) AND (intake)) OR “caloric restriction*” OR (diet* AND (quality OR intake)) OR “metabolic syndrome” OR “cardiovascular risk*” OR “cardiovascular dis*” OR cardiometabolic OR “glucose intoleran*” OR “glucose toleran*” OR “glucose dysregulation” OR igt OR “blood glucose” OR “blood sugar” OR “plasma glucose” OR “glucose level*” OR “glucose blood” OR “fasting glucose” OR hyperglycem* OR hyperglycaem* OR “Hb A1c” OR “HbA1c” OR “Hemoglobin A1c” OR diabet* OR “type 2 dm” OR “type II dm” OR NIDDM OR IDDM OR “insulin resistan*” OR “insulin sensitiv*” OR “homa-ir” OR homa2 OR hyperinsulin* OR prediabet* OR “blood pressure” OR “diastolic pressure” OR “systolic pressure” OR “pulse pressure” OR “arterial pressure” OR “aortic pressure” OR hypertensi* OR triglyceride* OR triacylglycerol* OR triacetylglycerol OR “triacetyl-glycerol” OR trielaidin OR trioleoylglycerol OR “trioleyl glycerol” OR “glycerol trioleate” OR “trioleate-glycerin” OR hypertriglyceridemi* OR cholesterol OR hdl OR vdl OR ldl OR idl OR “high density lipoprotein*” OR “hdl lipoprotein*” OR “low-density lipoprotein*” OR “very low lipoprotein*” OR obesit* OR obese* OR “body mass index” OR bmi OR “body weight” OR “body fat*” OR adiposit* OR “waist-hip ratio*” OR “waist to hip*” OR “waist circumference*” OR “weight change*” OR “weight gain*” OR “weight loss*” OR “weight reduction*” OR dyslipem* OR dyslipidem* OR dyslipaem* OR dyslipidaem* OR dyslipoprotein* OR hyperlipemi* OR hyperlipid* OR lipidemi* OR lipidaemi* OR lipemi* OR hypercholesterolemi* OR hypercholesteremi* OR hyperlipoproteinemi* OR hypoprebetalipoproteinemi*) OR KW ((food AND (quality OR purchas* OR consumption OR consuming OR intake)) OR ((energy OR nutrient* OR habitual OR diet OR dietary OR kcal OR kilocal* OR calories OR calory OR caloric OR food) AND (intake)) OR “caloric restriction*” OR (diet* AND (quality OR intake)) OR “metabolic syndrome” OR “cardiovascular risk*” OR “cardiovascular dis*” OR cardiometabolic OR “glucose intoleran*” OR “glucose toleran*” OR “glucose dysregulation” OR igt OR “blood glucose” OR “blood sugar” OR “plasma glucose” OR “glucose level*” OR “glucose blood” OR “fasting glucose” OR hyperglycem* OR hyperglycaem* OR “Hb A1c” OR “HbA1c” OR “Hemoglobin A1c” OR diabet* OR “type 2 dm” OR “type II dm” OR NIDDM OR IDDM OR “insulin resistan*” OR “insulin sensitiv*” OR “homa-ir” OR homa2 OR hyperinsulin* OR prediabet* OR “blood pressure” OR “diastolic pressure” OR “systolic pressure” OR “pulse pressure” OR “arterial pressure” OR “aortic pressure” OR hypertensi* OR triglyceride* OR triacylglycerol* OR triacetylglycerol OR “triacetyl-glycerol” OR trielaidin OR trioleoylglycerol OR “trioleyl glycerol” OR “glycerol trioleate” OR “trioleate-glycerin” OR hypertriglyceridemi* OR cholesterol OR hdl OR vdl OR ldl OR idl OR “high density lipoprotein*” OR “hdl lipoprotein*” OR “low-density lipoprotein*” OR “very low lipoprotein*” OR obesit* OR obese* OR “body mass index” OR bmi OR “body weight” OR “body fat*” OR adiposit* OR “waist-hip ratio*” OR “waist to hip*” OR “waist circumference*” OR “weight change*” OR “weight gain*” OR “weight loss*” OR “weight reduction*” OR dyslipem* OR dyslipidem* OR dyslipaem* OR dyslipidaem* OR dyslipoprotein* OR hyperlipemi* OR hyperlipid* OR lipidemi* OR lipidaemi* OR lipemi* OR hypercholesterolemi* OR hypercholesteremi* OR hyperlipoproteinemi* OR hypoprebetalipoproteinemi*) | 253,259 |
| S2 | DE ("Smartphone Use" OR "Spatial Navigation") OR TI ("geographic* information system*" OR "global positioning system*" OR gps OR (tracker* AND (route* OR location OR address)) OR "mobile phone*" OR "mobile telephone*" OR smartphone* OR "smart phone*" OR "ecological momentary assessment*" OR ema OR "geographical momentary assessment*" OR "activity spac*" OR "spatial epidemiology ") OR AB ("geographic* information system*" OR "global positioning system*" OR gps OR (tracker* AND (route* OR location OR address)) OR "mobile phone*" OR "mobile telephone*" OR smartphone* OR "smart phone*" OR "ecological momentary assessment*" OR ema OR "geographical momentary assessment*" OR "activity spac*" OR "spatial epidemiology ") OR KW ("geographic* information system*" OR "global positioning system*" OR gps OR (tracker* AND (route* OR location OR address)) OR "mobile phone*" OR "mobile telephone*" OR smartphone* OR "smart phone*" OR "ecological momentary assessment*" OR ema OR "geographical momentary assessment*" OR "activity spac*" OR "spatial epidemiology ") | 22,482 |
| S1 | DE ("Fast Food" OR "Retailing") OR TI (("food" AND ("environment*" OR "suppl*" OR "outlet*" OR "expos*" OR "desert*" OR "swamp*" OR "availab*" OR "access*" OR "proximit*" OR "densit*" OR "store*" OR "shop*")))) OR "foodscape" OR "restaurant*" OR "fastfood" OR "fast-food" OR "supermarket*" OR "retail") OR AB (("food" AND ("environment*" OR "suppl*" OR "outlet*" OR "expos*" OR "desert*" OR "swamp*" OR "availab*" OR "access*" OR "proximit*" OR "densit*" OR "store*" OR "shop*")))) OR "foodscape" OR "restaurant*" OR "fastfood" OR "fast-food" OR "supermarket*" OR "retail") OR KW (("food" AND ("environment*" OR "suppl*" OR "outlet*" OR "expos*" OR "desert*" OR "swamp*" OR "availab*" OR "access*" OR "proximit*" OR "densit*" OR "store*" OR "shop*")))) OR "foodscape" OR "restaurant*" OR "fastfood" OR "fast-food" OR "supermarket*" OR "retail") | 44,413 |

## **Supplementary Table 1e.** History and Search Details WEB OF SCIENCE Core Collection October 31, 2022

| **Set** | **Web of Science Core Collection Query** | **Results** |
| --- | --- | --- |
| **#4** | **#3 AND #2 AND #1** | 1,473 |
| **#3** | TS=(("food" AND ("quality" OR "purchas*" OR "consumption" OR "consuming" OR "intake")) OR (("energy" OR "nutrient*" OR "habitual" OR "diet" OR "dietary" OR "kcal" OR "kilocal*" OR "calories" OR "calory" OR "caloric" OR "food") AND ("intake")) OR "caloric restriction*" OR (diet* AND ("quality" OR "intake")) OR "metabolic syndrome" OR "cardiovascular risk*" OR "cardiovascular dis*" OR "cardiometabolic" OR "glucose intoleran*" OR "glucose toleran*" OR "glucose dysregulation" OR "igt" OR "blood glucose" OR "blood sugar" OR "plasma glucose" OR "glucose level*" OR "glucose blood" OR "fasting glucose" OR "hyperglycem*" OR "hyperglycaem*" OR "hb a1c" OR "hba1c" OR "hemoglobin a1c" OR "diabet*" OR "type 2 dm" OR "type ii dm" OR "niddm" OR "iddm" OR "insulin resistan*" OR "insulin sensitiv*" OR "homa-ir" OR "homa2" OR "hyperinsulin*" OR "prediabet*" OR "blood pressure" OR "diastolic pressure" OR "systolic pressure" OR "pulse pressure" OR "arterial pressure" OR "aortic pressure" OR "hypertensi*" OR "triglyceride*" OR "triacylglycerol*" OR "triacetylglycerol" OR "triacetyl-glycerol" OR "trielaidin" OR "trioleoylglycerol" OR "trioleyl glycerol" OR "glycerol trioleate" OR "trioleate-glycerin" OR "hypertriglyceridemi*" OR "cholesterol" OR "hdl" OR "vdl" OR "ldl" OR "idl" OR "high density lipoprotein*" OR "hdl lipoprotein*" OR "low-density lipoprotein*" OR "very low lipoprotein*" OR "obesit*" OR "obese*" OR "body mass index" OR "bmi" OR "body weight" OR "body fat*" OR "adiposit*" OR "waist-hip ratio*" OR "waist to hip*" OR "waist circumference*" OR "weight change*" OR "weight gain*" OR "weight loss*" OR "weight reduction*" OR "dyslipem*" OR "dyslipidem*" OR "dyslipaem*" OR "dyslipidaem*" OR "dyslipoprotein*" OR "hyperlipemi*" OR "hyperlipid*" OR "lipidemi*" OR "lipidaemi*" OR "lipemi*" OR "hypercholesterolemi*" OR "hypercholesteremi*" OR "hyperlipoproteinemi*" OR "hypoprebetalipoproteinemi*") | 3,342,538 |
| **#2** | TS= ("geographic* information system*" OR "global positioning system*" OR gps OR (tracker* AND (route* OR location OR address)) OR "mobile phone*" OR "mobile telephone*" OR smartphone* OR "smart phone*" OR "ecological momentary assessment*" OR "ema" OR "geographical momentary assessment*" OR "activity spac*" OR "spatial epidemiology ") | 206,421 |
| **#1** | TS= (("food" AND ("environment*" OR "suppl*" OR "outlet*" OR "expos*" OR "desert*" OR "swamp*" OR "availab*" OR "access*" OR "proximit*" OR "densit*" OR "store*" OR "shop*")) OR "foodscape" OR "restaurant*" OR "fastfood" OR "fast-food" OR "supermarket*" OR "retail") | **462,982** |

## **Additional file 1: Table S1f**. History and Search Details SCOPUS October 31, 2022

| **Set** | **Scopus Query** | **Results** |
| --- | --- | --- |
| **#4** | #1 AND #2 AND #3 | 2,069 |
| **#3** | TITLE-ABS-KEY (({food} AND ({quality} OR "purchas*" OR {consumption} OR {consuming} OR {intake})) OR (({energy} OR "nutrient*" OR {habitual} OR (55) OR {dietary} OR {kcal} OR "kilocal*" OR {calories} OR {calory} OR {caloric} OR {food}) AND {intake}) OR (diet* AND ({quality} OR {intake})) OR "caloric restriction*" OR "metabolic syndrome" OR "cardiovascular risk*" OR "cardiovascular dis*" OR "cardiometabolic" OR "glucose intoleran*" OR "glucose toleran*" OR "glucose dysregulation" OR "igt" OR "blood glucose" OR "blood sugar" OR "plasma glucose" OR "glucose level*" OR "glucose blood" OR "fasting glucose" OR "hyperglycem*" OR "hyperglycaem*" OR {hb a1c} OR {hba1c} OR {hemoglobin a1c} OR "diabet*" OR {type 2 dm} OR {type ii dm} OR {niddm} OR {iddm} OR "insulin resistan*" OR "insulin sensitiv*" OR {homa-ir} OR {homa2} OR "hyperinsulin*" OR "prediabet*" OR "blood pressure" OR "diastolic pressure" OR "systolic pressure" OR "pulse pressure" OR "arterial pressure" OR "aortic pressure" OR "hypertensi*" OR "triglyceride*" OR "triacylglycerol*" OR "triacetylglycerol" OR "triacetyl-glycerol" OR "trielaidin" OR "trioleoylglycerol" OR "trioleyl glycerol" OR "glycerol trioleate" OR "trioleate-glycerin" OR "hypertriglyceridemi*" OR "cholesterol" OR "hdl" OR "vdl" OR {ldl} OR {idl} OR "high density lipoprotein*" OR "hdl lipoprotein*" OR "low-density lipoprotein*" OR "very low lipoprotein*" OR "obesit*" OR "obese*" OR "body mass index" OR {bmi} OR "body weight" OR "body fat*" OR "adiposit*" OR "waist-hip ratio*" OR "waist to hip*" OR "waist circumference*" OR "weight change*" OR "weight gain*" OR "weight loss*" OR "weight reduction*" OR "dyslipem*" OR "dyslipidem*" OR "dyslipaem*" OR "dyslipidaem*" OR "dyslipoprotein*" OR "hyperlipemi*" OR "hyperlipid*" OR "lipidemi*" OR "lipidaemi*" OR "lipemi*" OR "hypercholesterolemi*" OR "hypercholesteremi*" OR "hyperlipoproteinemi*" OR "hypoprebetalipoproteinemi*") | 4,811,429 |
| **#2** | TITLE-ABS-KEY ("geographic* information system*" OR "global positioning system*" OR gps OR (tracker* AND (route* OR location OR address)) OR "mobile phone*" OR "mobile telephone*" OR smartphone* OR "smart phone*" OR "ecological momentary assessment*" OR "ema" OR "geographical momentary assessment*" OR "activity spac*" OR "spatial epidemiology ") | 447,642 |
| **#1** | TITLE-ABS-KEY(("food" AND ("environment*" OR "suppl*" OR "outlet*" OR "expos*" OR "desert*" OR "swamp*" OR "availab*" OR "access*" OR "proximit*" OR "densit*" OR "store*" OR "shop*")) OR "foodscape" OR "restaurant*" OR "fastfood" OR "fast-food" OR "supermarket*" OR "retail") | 790,380 |

## **Additional file 1: Table S1g.** History and Search Details IBSS October 31, 2022

| **Set** | **IBSS (ProQuest) Query** | **Results** |
| --- | --- | --- |
| #4 | #1 AND #2 AND #3 | 103 |
| #3 | MAINSUBJECT.EXACT("Food quality" OR "Diet" OR "Nutrition" OR "Metabolic syndrome" OR "Metabolism" OR "Diabetes" OR "Hyperglycemia" OR "Obesity" OR "Glycemic index" OR "Low density lipoprotein" OR "Body mass index" OR "Lipoproteins" OR "Insulin resistance" OR "Blood pressure" OR "Cholesterol" OR "Hypertension" OR "Cardiovascular disease") OR TITLE,ABSTRACT(("food" AND ("quality" OR "purchas*" OR "consumption" OR "consuming" OR "intake")) OR (("energy" OR "nutrient*" OR "habitual" OR "diet" OR "dietary" OR "kcal" OR "kilocal*" OR "calories" OR "calory" OR "caloric" OR "food") AND ("intake")) OR "caloric restriction*" OR (diet* AND ("quality" OR "intake")) OR "metabolic syndrome" OR "cardiovascular risk*" OR "cardiovascular dis*" OR "cardiometabolic" OR "glucose intoleran*" OR "glucose toleran*" OR "glucose dysregulation" OR "igt" OR "blood glucose" OR "blood sugar" OR "plasma glucose" OR "glucose level*" OR "glucose blood" OR "fasting glucose" OR "hyperglycem*" OR "hyperglycaem*" OR "hb a1c" OR "hba1c" OR "hemoglobin a1c" OR "diabet*" OR "type 2 dm" OR "type ii dm" OR "niddm" OR "iddm" OR "insulin resistan*" OR "insulin sensitiv*" OR "homa-ir" OR "homa2" OR "hyperinsulin*" OR "prediabet*" OR "blood pressure" OR "diastolic pressure" OR "systolic pressure" OR "pulse pressure" OR "arterial pressure" OR "aortic pressure" OR "hypertensi*" OR "triglyceride*" OR "triacylglycerol*" OR "triacetylglycerol" OR "triacetyl-glycerol" OR "trielaidin" OR "trioleoylglycerol" OR "trioleyl glycerol" OR "glycerol trioleate" OR "trioleate-glycerin" OR "hypertriglyceridemi*" OR "cholesterol" OR "hdl" OR "vdl" OR "ldl" OR "idl" OR "high density lipoprotein*" OR "hdl lipoprotein*" OR "low-density lipoprotein*" OR "very low lipoprotein*" OR "obesit*" OR "obese*" OR "body mass index" OR "bmi" OR "body weight" OR "body fat*" OR "adiposit*" OR "waist-hip ratio*" OR "waist to hip*" OR "waist circumference*" OR "weight change*" OR "weight gain*" OR "weight loss*" OR "weight reduction*" OR "dyslipem*" OR "dyslipidem*" OR "dyslipaem*" OR "dyslipidaem*" OR "dyslipoprotein*" OR "hyperlipemi*" OR "hyperlipid*" OR "lipidemi*" OR "lipidaemi*" OR "lipemi*" OR "hypercholesterolemi*" OR "hypercholesteremi*" OR "hyperlipoproteinemi*" OR "hypoprebetalipoproteinemi*") | 37,592 |
| #2 | MAINSUBJECT.EXACT("Global positioning systems--GPS" OR "Geographic information systems" OR "Smartphones" OR "Spatial analysis") OR TITLE,ABSTRACT("geographic* information system*" OR "global positioning system*" OR gps OR (tracker* AND (route* OR location OR address)) OR "mobile phone*" OR "mobile telephone*" OR smartphone* OR "smart phone*" OR "ecological momentary assessment*" OR "ema" OR "geographical momentary assessment*" OR "activity spac*" OR "spatial epidemiology ") | 20,998 |
| #1 | MAINSUBJECT.EXACT("Food supply" OR "Food deserts" OR "Food stamps" OR "Fast food" OR "Fast food industry" OR "Restaurants") OR TITLE,ABSTRACT(("food" AND ("environment*" OR "suppl*" OR "outlet*" OR "expos*" OR "desert*" OR "swamp*" OR "availab*" OR "access*" OR "proximit*" OR "densit*" OR "store*" OR "shop*")) OR "foodscape" OR "restaurant*" OR "fastfood" OR "fast-food" OR "supermarket*" OR "retail") | 39,035 |

## **Google Scholar query**

food|restaurant|fastfood|supermarket|retail+gps|tracker|phone|ema|gma|spacing|spatial|geographic+food|intake|caloric|metabolic|cardiovascular|glucose|igt|hyperglycemia|hba1c|diabetes|insulin|blood pressure|hypertension|hdl|vdl|ldl|idl|obesity|bmi|weight|fat|waist

**Additional file 1: Table S2.** Data extraction table

| **Ref.** | **Country/location where study was conducted** | **% female** | **Age range**  **(years)** | **Study population** | **Sample size** | **Study design** | **Methods of exposure data collection (e.g., GPS trackers, mobile devices)** | **Reported loss of signal from GPS devices** | **How was the food environment assessed based on GPS data? (e.g., GPS points buffered, of food outlets along each trip)** | **Tracking duration, GPS sampling frequency** | **Temporal aspects taken into account (i.e., temporal aspects of human mobility or food environment (e.g., opening hours))** | **Type and distribution of outcome: continuous/dichotomous, type of diet or diet and cardiometabolic related health outcome** |
| --- | --- | --- | --- | --- | --- | --- | --- | --- | --- | --- | --- | --- |
| Zenk et al. (2011) (1) | Detroit (USA) | 75.0 | <45, 45-64, >64 | Participants were recruited from a 6-year follow-up study as part of the The Detroit Activity Space Environments Study (DASES) Study. | 120 | Cross-sectional | GPS device: Foretrex 201 | Reported | **Food environment data source**: lists of food store places from the government; **Food environment assessed**: fast food outlet density and the number of chain full-service grocery stores or supercenters; **GPS-based food exposure measures**: one standard deviation ellipse on GPS points and 0.5 mile daily path area on GPS points. | Tracking duration: 7 days. Frequency GPS sampling: 30 second intervals | Not reported | Set of Food frequency questionnaire items reporting on saturated fat intake, fruit and vegetable intake, and whole grain intake (validation of FFQ not reported) |
| Widener et al. 2018 (2) | Toronto, Montreal, Vancouver, Edmonton, and Halifax (Canada) | 66.5 | 16-30 | Participants were recruited by trained research assistants using in-person intercept sampling, from a sample of sites stratified by region/neighborhood and site type. | 496 | Cross-sectional | Smartphone: CFSMobile app | Not reported | **Food environment data source:** 2016 DMTI business directory; **Food environment assessed**: the number of grocery stores, convenience stores, fruit and vegetable markets, limited-service restaurants and ‘all food retailers’; **GPS-based food exposure measures**: regular activity space locations (defined as the top 1% and 10% of the time-weighted kernel density estimate surface). | Tracking duration: 7 days. Frequency GPS sampling: not reported | Not reported | Webform (Counts of food purchasing). |
| Wang et al. (2018) (3) | Franklin County (USA) | 60.8 | 18-65+ | Participants were recruited in person in selected public parks and neighborhoods surrounding these parks following household interviews. | 46 | Cross-sectional | GPS device | Reported and inserted or considered as missing | **Food environment data source**: lists of food store places from the government; **Food environment assessed**: environmental context exposure index and density of fast-food restaurants, convenience stores, meat markets, pizzerias, bakeries, and candy and nut stores; **GPS-based food exposure measures**: 100 m 3-D GPS trajectory buffers (geolocation and time considered), 100 m GPS trajectory buffers, standard deviation ellipses with one or two standard deviations on GPS points, and minimum convex polysons of GPS points. | Tracking duration 3 weeks. Frequency GPS sampling: every minute | Opening hours of food stores were taken into account. | BMI was calculated by dividing the subject's weight (kg) with height in meters squared (m2) (not reported whether it was self-reported or not). |
| Wray et al. (2021) (4) | London (Canada) | 63.6 | 13-18 | Participant data for this analysis are sources from a large multi-year intervention study of adolescents. Study is targeted to high school students. | 154 | Cross-sectional | Smartphone: SmartAPPetite | Not reported | **Food environment data source**: lists of food store places from the government; **Food environment assessed**: standardized count of logged GPS points within the buffer of retail food outlets's ads (quick service, restaurants, grocery, and variety) ; **GPS-based food exposure measures**: duclidean buffers around outlets (150 m), billboards (150 m), bus shelters (75 m), and street posters (75 m). | Tracking duration: 12 weeks. Frequency GPS sampling: every 120 seconds or when a user enters the geofenced area surrounding a retail food outlet | Not reported | Food purchasing, categorical (quick service purchases, restaurants purchases, grocery purchases, variety purchases, all types purchases). |
| Tamura et al. (2018) (5) | New York City (USA) | 51.9 | 18+ | Participants were recruited from the NYC Low-Income Housing, Neighborhoods and Health Study. Low-income housing residents in NYC were recruited through outreach activities, such as handing out flyers around public housing developments in NYC, circulating flyers through community-based organizations, or via social networks. | 102 | Cross-sectional | GPS device (Qstarz BT-Q1000XT GPS, Qstarz) | Reported | **Food environment data source**: lists of food store places from the government; **Food environment assessed**: Density of fast-food restaurants, wait-service restaurants, corner stores, grocery stores and supermarkets; **GPS-based food exposure measures**: 200 m and 400 m daily mobility path on GPS points. | Tracking duration: 7 days. Frequency GPS sampling: every 30 seconds | Not reported | Tanita 351 scale was used to measure participants' heights and weights, which were then used to compute BMI. Blood pressure was measured for 15-40 seconds SBP and DBP (based on mmHg) was assess with a Welch Allyn Vital Signs 300 monitor. |
| Liu et al. (2020) (6) | Toronto, Montreal, Vancouver, Edmonton, and Halifax (Canada) | 65.0 | 16-30 | Data come from the Canada Food Study, focused on young adults | 591 | Cross-sectional | Smartphone: Itinerium | Not reported | **Food environment data source**: lists of food store places from opensource platform (OpenStreetMap); **Food environment assessed**: the number of fast food outlets, and the proportion of the sum of the number of fast food outlets, supermarkets, green groceries, and convenience stores within each activity space; **GPS-based food exposure measures**: 500 m, 1000 m, and 1500 m circular buffers on activity locations . | Tracking duration: 7 days. Frequency GPS sampling: frequency not reported | Time-weighted exposure was taken into account by calculating the proportion of the time a participant spent at each activity location in the total time spent in all activity space. | Self-reported fast-food intake |
| Burgoine et al. (2015) (7) | Mebane, North Carolina (USA) | 48.9 | 5-11 | Children and their parents were recruited through three schools in each town. | 94 | Cross-sectional | GPS device (Qstarz BT-Q1000XT GPS, Qstarz) | Reported | Food environment data source: lists of food store places from a commercial company (Reference USA); Food environment assessed: Density of takeaway food outlets and all food outlets; GPS-based food exposure measures: 100 m GPS route buffer. | Tracking duration: 7 days. Frequency GPS sampling: every 60 seconds | Not reported | Height and weight were measured by trained research staff (using a Seca 124 Portable stadiometer and a Tanita BWB-800 portable scale, respectively), and age-specific BMI z-scores calculated relative to growth charts from the US CDC. |
| Elliston et al. (2020) (8) | Tasmania (Australia) | 71.0 | 18+ | Participants were recruited by looking at everyday food choices through social media advertising and a university staff newsletter in Tasmania. | 72 | Cross-sectional | Smartphone | Not reported | **Food environment data source**: lists of food store places from the government; **Food environment assessed**: The number of food outlets; **GPS-based food exposure measures**: 50 m GPS point buffer. | Tracking duration: 2 weeks. Frequency GPS sampling: once the user report food intake | Not reported | Eating/Non-eating and food intake (collected by food reports and EMA measures: prompts, 4-5 times a day during a period of 2 weeks. |
| Ghosh Roy et al. (2019) (9) | Chicago (USA) | 100 | 25-65 | Participants were recruited from community sites and an urban public university. | 79 | Cross-sectional | GPS device (Qstarz BT-Q1000XT GPS, Qstarz) | Not reported | **Food environment data source**: lists of food store places from a commercial company (Dun & Bradstreet); **Food environment assessed:** The number of fast food restaurants and convenience stores**; GPS-based food exposure measures:** 400 m GPS route buffer. | Tracking duration: 7 days. Frequency GPS sampling: every minute. | Not reported | Snack food item and sweetened beverage intake dichotomized into non or one (0) or more than one (1). |
| Gustafson et al. (2013) (10) | Lexington (USA) | 58.0 | 18+ | Participants were recruitment via mailed flyers describing the research and contact information. | 121 | Cross-sectional | GPS device (Qstarz BT-Q1000XT Travel Recorder) | Not reported | **Food environment data source**: lists of food store places from a commercial company (InfoUSA); **Food environment assessed:** the retail food environment index as a ratio of healthy (Supermarkets/grocery stores, farmers’ markets, and produce stands) relative to unhealthy (supercenters, convenience stores, fast-food restaurants, and gas stations with convenience stores, or less healthy venues) food venues**; GPS-based food exposure measures**: 0.5 mile GPS route buffer. | Tracking duration: 3 days (2 weekdays and 1 weekend day). Frequency GPS sampling: not reported. | Not reported | Based on the NEMS-S protocol, availability, price, and quality of food were collected for 15 food categories (fruit, vegetables, milk, cheese, meat, baked goods, chips, beverages, canned items, cereal, desserts, prepared food items, snack foods, frozen meals, and beans) and 55 unique food items were assessed. |
| Shearer et al. (2015) (11) | Halifax (Canada) | Not reported | 12-16 | Particpants were recruited from six schools in the Halifax Regional School Board in Nova Scotia, Canada. | 380 | Cross-sectional | GPS device (20 channel EM-408 SiRFstar III chipset GSP receiver) | Reported and fixed | **Food environment data source:** lists of food store places from a commercial company (DMTI); Food environment assessed: The number of fast food (major fast food chains with a focus on take-away meals), restaurants (all other types), grocery and convenience stores, and average distances to every accessible and actually visited food locations within GPS route buffers from a participant's home and school origin; GPS-based food exposure measures: 50 m GPS route buffer. | Tracking duration: 7 days. Frequency GPS sampling: every second | Not reported | diet quality index score ranging from 0 to 100 with higher scores reflecting better diet quality. |
| Seto et al. (2016) (12) | Kunming (China) | 66.7 | 18-31 | Participants were recruited among students at the Kunming Medical University | 12 | Cross-sectional | Smartphone: CalFit Chi and Dong | Not reported | **Food environment data source:** lists of food store places from Google Maps; Food environment assessed: average number of bakery, bar, cafe, convenience store, food, grocery or supermarket, liquor store, meal delivery, meal takeaway, and restaurant; **GPS-based food exposure measures**: 250 m circular buffers on activity locations. | Tracking duration: 6 days. Frequency GPS sampling: every 10 seconds | Not reported | Portion size was recorded with video. Later, two trained dietitians familiar with local diets review the contents of the videos, and code the portion sizes and food groups associated with each food consumed. Subjects’ diet recordings were coded by both dietitians in order to assess inter-rater reliability. |
| Christian et al. (2012) (13) | Lexington-Fayette County (USA) | 56.4 | 18-65 | Participant were recruitment via mailed flyers and announcements at neighborhood association meetings. | 121 | Cross-sectional | GPS device (Qstarz BT-Q1000XT Travel Recorder) | Not reported | **Food environment data source**: lists of food store places from the government; **Food environment assessed**: the retail food environment index calculated as the sum of fast food restaurants plus convenience stores, divided by the sum of supermarkets plus fruit/vegetable markets, the proportion and density of limited-service outlets and supermarket; **GPS-based food exposure measures**: 0.5 mile GPS route buffer. | Tracking duration: 3 days. Frequency GPS sampling: every 3 seconds | Not reported | Continues dietary intake of added sugar, red meat, fried potatoes, fruits and vegetables, whole grains. Weight status categorized into overweight and obese. |
| Sadler et al. (2016) (14) | Southwestern Ontario (Canada) | 58.7 | 9-13 | Participants were recruited as part of the Spatial Temporal Environment and Activity Monitoring (STEAM) Project from communities in southwestern Ontario. | 654 | Cross-sectional | GPS device | Not reported | **Food environment data source**: lists of food store places from public health inspectors; **Food environment assessed**: the number of minutes during which a child was exposed (i.e. within 50 m) to fast food, variety stores, pizza places or ice cream shops (ranges from 0 sec to 350 min); **GPS-based food exposure measures**: 50 m buffers on food outlets. | Tracking duration 2 weeks. Frequency GPS sampling: every second | Not reported | Junk food purchasing outcome (binary), indicating whether junk food was purchased or not on the trip. Junk food was considered unhealthy food items purchased from fast food or variety stores, pizza places and ice cream shops. |

**Additional file 1: Table S3.** Quality assessment according to the Newcastle-Ottawa Scale (NOS) and included items from Cetateanu et al.

| First author (Year) | Selection Comparability Outcome | | | | | | | GPS exposure assessment | |  |  |  |  |
| --- | --- | --- | --- | --- | --- | --- | --- | --- | --- | --- | --- | --- | --- |
|  | **Representativeness of the samples** | **Sample size calculation** | **Non-respondents** | **Ascertainment of the exposure (risk factor)** | **Adjustment for confounders** | **Assessment of the outcome** | **Statistical test** | **Recording period** | **Assessment of variety of food outlet types** | **Positional accuracy of the reported device** | **GPS data quality discussed** | **Total** |  |
| Widener (2018)^a^ | 0 | 1 | 0 | 1 | 1 | 1 | 1 | 2 | 3 | 0 | 0 | 10 |  |
| Wray (2021)^b^ | 0 | 0 | 0 | 1 | 1 | 1 | 1 | 2 | 3 | 0 | 1 | 10 |  |
| Sadler (2016)^c^ | 0 | 1 | 0 | 1 | 2 | 1 | 1 | 2 | 2 | 0 | 0 | 10 |  |
| Shearer (2015)^d^ | 0 | 1 | 0 | 1 | 2 | 1 | 0 | 2 | 2 | 1 | 1 | 11 |  |
| Zenk (2011)^e^ | 1 | 0 | 0 | 1 | 1 | 1 | 0 | 2 | 2 | 0 | 1 | 9 |  |
| Elliston (2020)^f^ | 1 | 1 | 0 | 1 | 0 | 1 | 0 | 2 | 1 | 0 | 0 | 7 |  |
| Gustafson (2013)^g^ | 1 | 0 | 0 | 1 | 1 | 1 | 1 | 1 | 3 | 0 | 0 | 9 |  |
| Ghosh Roy (2019)^h^ | 0 | 1 | 0 | 1 | 1 | 1 | 1 | 2 | 2 | 0 | 0 | 9 |  |
| Seto (2016)^i^ | 1 | 1 | 0 | 1 | 2 | 1 | 1 | 2 | 3 | 0 | 0 | 12 |  |
| Liu (2020)^j^ | 0 | 0 | 1 | 1 | 1 | 1 | 1 | 2 | 2 | 0 | 1 | 10 |  |
| Christian (2012)^k^ | 1 | 0 | 1 | 1 | 1 | 1 | 1 | 1 | 2 | 0 | 0 | 9 |  |
| Burgoine (2015)^l^ | 0 | 0 | 0 | 1 | 1 | 2 | 1 | 2 | 2 | 0 | 1 | 10 |  |
| Tamura (2018)^m^ | 0 | 1 | 1 | 1 | 1 | 2 | 1 | 2 | 3 | 0 | 1 | 13 |  |
| Wang. (2018)^n^ | 1 | 0 | 0 | 1 | 1 | 1 | 1 | 2 | 3 | 0 | 1 | 11 |  |

**Additional Information 1**

List of supplementary citations for referencing the studies included in the systematic review.

a Widener MJ, Minaker LM, Reid JL *et al.* (2018) Activity space-based measures of the food environment and their relationships to food purchasing behaviours for young urban adults in Canada. *Public Health Nutr* 21, 2103-2116.

b Wray A, Martin G, Doherty S *et al.* (2021) Analyzing differences between spatial exposure estimation methods: A case study of outdoor food and beverage advertising in London, Canada. *Health Place*, 102641.

c Sadler RC, Clark AF, Wilk P *et al.* (2016) Using GPS and activity tracking to reveal the influence of adolescents' food environment exposure on junk food purchasing. *Can J Public Health* 107, 5346.

d Shearer C, Rainham D, Blanchard C *et al.* (2015) Measuring food availability and accessibility among adolescents: Moving beyond the neighbourhood boundary. *Soc Sci Med* 133, 322-330.

e Zenk SN, Schulz AJ, Matthews SA *et al.* (2011) Activity space environment and dietary and physical activity behaviors: a pilot study. *Health Place* 17, 1150-1161.

f Elliston KG, Schuz B, Albion T *et al.* (2020) Comparison of Geographic Information System and Subjective Assessments of Momentary Food Environments as Predictors of Food Intake: An Ecological Momentary Assessment Study. *JMIR Mhealth Uhealth* 8, e15948.

g Gustafson A, Christian JW, Lewis S *et al.* (2013) Food venue choice, consumer food environment, but not food venue availability within daily travel patterns are associated with dietary intake among adults, Lexington Kentucky 2011. *Nutr J* 12, 17.

h Ghosh Roy P, Jones KK, Martyn-Nemeth P *et al.* (2019) Contextual correlates of energy-dense snack food and sweetened beverage intake across the day in African American women: An application of ecological momentary assessment. *Appetite* 132, 73-81.

i Seto E, Hua J, Wu L *et al.* (2016) Models of Individual Dietary Behavior Based on Smartphone Data: The Influence of Routine, Physical Activity, Emotion, and Food Environment. *PLoS One* 11, e0153085.

j Liu B, Widener M, Burgoine T *et al.* (2020) Association between time-weighted activity space-based exposures to fast food outlets and fast food consumption among young adults in urban Canada. *Int J Behav Nutr Phys Act* 17, 62.

k Christian WJ (2012) Using geospatial technologies to explore activity-based retail food environments. *Spat Spatiotemporal Epidemiol* 3, 287-295.

l Burgoine T, Jones AP, Namenek Brouwer RJ et al. (2015) Associations between BMI and home, school and route environmental exposures estimated using GPS and GIS: do we see evidence of selective daily mobility bias in children? Int J Health Geogr 14, 8.

m Tamura K, Elbel B, Athens JK *et al.* (2018) Assessments of residential and global positioning system activity space for food environments, body mass index and blood pressure among low-income housing residents in New York City. *Geospat Health* 13.

n Wang J & Kwan MP (2018) An Analytical Framework for Integrating the Spatiotemporal Dynamics of Environmental Context and Individual Mobility in Exposure Assessment: A Study on the Relationship between Food Environment Exposures and Body Weight. *Int J Environ Res Public Health* 15.
